# The Failure Index as Marker of the Neural Health Profile in MED-EL CI Users: A Retrospective Analysis

**DOI:** 10.1177/23312165261477505

**Published:** 2026-08-20

**Authors:** Wiebke Konerding, Cornelia Batsoulis, Peter Baumhoff, Heval Benav, Lutz Gärtner, Annette Günther, Onhintz de Olano Dieterich, Daniel Schurzig, Stefan Strahl, Jochen Tillein, Sarah Vormelcher, Andreas Büchner, Carolyn Garnham, Andrej Kral

**Affiliations:** 1 Department of Experimental Otology, 9177 Hannover Medical School, Hannover, Germany; 2 MED-EL Research Center, 84941 MED-EL Medical Electronics GmbH, Hannover, Germany; 3 Research & Development, 84941 MED-EL Medical Electronics GmbH, Innsbruck, Austria; 4 Department of Otolaryngology, 9177 Hannover Medical School, Hannover, Germany; 5 Clinics of Otolaryngology, Head and Neck Surgery, J.W. Goethe University, Frankfurt, Germany

**Keywords:** neural health marker, electrically-evoked compound action potential, objective measure, cochlear implant

## Abstract

Cochlear implants (CIs) enable hearing via direct electrical stimulation of the spiral ganglion neurons (SGN). Outcomes with a CI depend, in part, on the number and excitability of the SGNs. In an animal model, we introduced the Failure Index (FI) as an electrically-evoked compound action potential (eCAP)-derived marker of cochlear health. The FI informs about the presence, site, and size of SGN lesions. Here, we translated the FI to clinical recordings from human MED-EL CI users. In this retrospective study, we included patient data recorded between 2016 and 2024 from 199 ears with postlingual hearing loss and 79 ears with prelingual hearing loss. The averaged FI values over all contacts of the array were stable within the analysis period (3^rd^ month to 1^st^ year postoperatively). The FI increased with age and was elevated in etiologies associated with higher SGN loss. Using 3D reconstruction from cone-beam computed tomography scans, we confirmed that the FI was independent of electrode-to-modiolus distance (0.1–2.5 mm). The FI showed individual patterns along the array, with maxima usually found at basal contacts, corresponding to reduced neural health at high-frequency cochlear regions. In a selected group of postlingual-deaf ears, we found higher correlation coefficients between speech-perception scores and the FI compared to other eCAP-derived markers (i.e., threshold, slope, and amplitude). These results were in line with the hypothesis that the FI may serve as a clinical tool to identify implanted ears with reduced neural health and to identify contacts stimulating areas of reduced SGN survival and integrity.

## Introduction

Cochlear implants (CI) enable speech understanding in users with hearing loss, including adults with postlingual hearing loss (e.g., Sargsyan et al., 2021) and early-implanted children with prelingual hearing loss (e.g., Illg et al., 2024). However, speech understanding outcomes vary substantially across users (Boisvert et al., 2020; Illg et al., 2024). Understanding speech in background noise remains a challenge for many CI users (for review see Henry et al., 2021). Predictive factors are scarce, and explain only part of the outcome variability (Demyanchuk et al., 2025; Günther et al., 2025; Zhao et al., 2020). Besides cognitive-linguistic factors (e.g., Zhan et al., 2020), the factors that have been repeatedly found to influence speech understanding are age and duration of hearing loss (e.g., Heutink et al., 2021; O'Donoghue et al., 2000).

One important peripheral factor is neural health, which we define in the context of CI use as all factors that enable the efficient transmission of electrical current into neural activation. These include spiral ganglion neuron (SGN) integrity (i.e., cell count, number of peripheral processes, and myelination of peripheral and central axon), as well as metabolic and genetic factors (Zamaninezhad et al., 2023). As it is not possible to assess the survival of the targeted human SGN *in vivo* a reliable electrophysiologic marker is needed (Zhan et al., 2021).

A neural health marker would facilitate prediction of the best possible outcomes with a CI, stratify diagnostic findings to identify patients with additional needs for rehabilitation, and could be used to tailor audio processor programming to individual requirements (for review see Lien et al., 2025). Functional measures of neural health are often derived from the electrically-evoked compound action potential (eCAP) that can be recorded non-invasively via the CI (for review see Schvartz-Leyzac et al., 2023).

In humans, a common way to assess the potential utility of a measure as a neural health marker is to demonstrate a significant correlation with speech understanding scores. So far, attempts have led to mixed results, with eCAP threshold in monopolar stimulation mode usually failing to show a significant correlation with speech outcomes, though significant correlations have been found with focused thresholds (Arjmandi et al., 2022; Peng et al., 2025). Other proposed markers usually explained less than 30% of the variance in speech understanding (for review see Dawson et al., 2025; Schvartz-Leyzac et al., 2023; Schvartz-Leyzac et al., 2025). For different electrophysiological markers, the correlation was reported to be stronger for speech perception in noise than in quiet listening (Schvartz-Leyzac & Pfingst, 2018; Skidmore et al. 2022, 2023).

Several studies have reported that high variability of focused thresholds across electrode contacts can negatively impact speech recognition (DeVries et al., 2016; Pfingst et al., 2004; Zhou & Pfingst, 2014). Pfingst and colleagues (2004) reported that only across-site variation in thresholds, but not mean thresholds, were correlated with speech recognition. The proposed mechanism is that contacts adjacent to regions of low neural health require higher stimulation levels to achieve a perceived loudness similar to that of other CI contacts in healthier cochlear regions. However, neighbouring regions with better neural health will also be stimulated by these higher stimulation levels. This leads to spectral smearing which distorts the place-pitch code and increases variation in the number of neurons activated by each contact (Fu & Nogaki, 2005; Pfingst et al., 2004). Thus, compensating for focal regions of reduced SGN integrity will be more detrimental for speech understanding than compensating for homogeneous degeneration over the entire cochlea when the degeneration is of the same average magnitude.

To optimize information flow through the implant, the audio processor can be re-programmed by adjusting the frequency allocation, or simply by deactivating single contacts (Lien et al., 2025). A recent review among audiologists reported that a majority believed that deactivating contacts close to areas of reduced neural health would improve speech understanding (Sander et al., 2023). However, the same study reported that selective deactivation is rarely performed during clinical routine, as guidelines for selecting which contacts to deactivate are currently missing (Sander et al., 2023). Thus, a neural health marker needs to be sensitive to spatially and functionally variable auditory nerve integrity along the cochlea (i.e., neural health profile; Genitsaridi et al., 2026).

We introduced a marker of the neural health profile, the Failure Index (FI), in an animal model (Konerding et al., 2025). In the guinea pig, the FI separated lesioned from non-lesioned ears at the overall median (i.e., grand median, FI_GM_), allowing an identification of reduced neural health at the individual level. Furthermore, the maximum FI value along the array corresponded to the contact closest to the lesion in 80% of cases (i.e., except for small lesion sizes). In the guinea pig, the FI was a more reliable predictor of SGN lesion size than other characteristics of the eCAP amplitude-growth function (AGF), such as threshold, slope, or maximal amplitude.

The FI is calculated as the input/output ratio at maximal activation, indicating the failure to efficiently transform electrical current into strong neural signals (i.e., synchronous activation). The rationale for calculating the ratio between stimulation current and response amplitude is a normalisation to input current, thus generating a comparable matrix between different contacts and ears. We hypothesise that this (partial) normalisation for current spread reduces the influence of distance to excitable structures. This contrasts with the posited dependence of threshold and amplitude on electrode-to-modiolar distance (EMD; Degen et al., 2020).

The FI significantly correlated with SGN loss in the animal lesion model (Konerding et al., 2025). However, we propose that it may be influenced by all factors of neural health, as these influence the magnitude of synchronous SGN excitation. Mathematically, the FI is the inverse of the slope through the origin point. In this way, the FI would reflect the increase in neural activation with increasing input, when disregarding any influences on threshold levels, such as spontaneous activity and measurement noise.

As a first step to assess the translational potential of the FI for human CI users, we retrospectively analysed clinical eCAP recordings (all from MED-EL devices). We assessed whether the results corresponded to hypotheses that are in line with the notion that the FI reflects the neural health profile. These were: 1) stability over several months post-surgery, 2) group differences (i.e., age and etiology), 3) correlation with speech perception outcomes, 4) individual profiles along the electrode array, and 5) independency from the electrode-to-modiolus distance.

## Material and Methods

### Subjects

For this retrospective cohort study, we selected 278 CI-implanted ears for analysis (initially N=402: inclusion and exclusion criteria, see below). These were from 253 CI users (median age: 54 years; 140 females; classification as per voluntary self-identification; see patient demographics in Appendix 1). The age was defined as the age at implantation, as all measurements were performed within the same time period from 3^rd^ month (M3) to 1^st^ year (Y1) post-surgery.

All included ears received a flexible electrode array of the FLEX series (MED-EL; details see Appendix 1). The array model was selected to match the individual cochlear anatomy. In cases of simultaneous bilateral implantation, both ears were included and were analysed separately. Exclusion criteria were (non-simultaneously implanted) second ears (n=91), atypical array types (i.e., n=6 short arrays (FLEX16 and FLEX20) and n=7 stiff electrode arrays), stem cell applications (n=6), and reimplanted ears (n=14). With the exception of the assessment of anatomical relations (see ‘Correlation with electrode-to-modiolus distance’), ears with partial array insertion were also excluded (n=49). Thus, a total of 199 ears with postlingual hearing loss and 79 ears with prelingual hearing loss were included in the final analysis.

For the analysis of differences based on age and etiology, we also excluded ears which had received short- or long-term local steroid application (n=16), as this treatment may alter age- and etiology-typical neural health profiles (Jin et al., 2009). To assess the correlations with outcome measures, we applied additional exclusion criteria to reduce the variability due to differences in language experience and number of contacts available for analysis (for details on selection of the reduced dataset see ‘Correlation with speech scores’). We also differentiated between CI-users with post- and prelingual hearing loss, where necessary, due to differences in the distributions of etiologies that impacted the outcome measure, as well as differences in overall speech understanding between these groups (details below).

The CI surgeries were performed at the Hannover Medical School (MHH) from August 16^th^ 2016 to February 2^nd^ 2024. The postsurgical data were collected during clinical visits at the German Hearing Center Hannover (a tertiary care centre, specialized in the treatment of hearing loss). Objective clinical and anatomical data were integrated into the CI database (for details see, Günther et al., 2025).

### ECAP Recordings

All eCAP recordings were performed during clinical visits between three months to one year post surgery. The response of the auditory nerve to CI stimulation was assessed by measuring the eCAP N1-P1 amplitude, using the automated continuous eCAP measurement function ‘AutoART’ (Strahl, 2018) of the MAESTRO fitting software (Version 7 or higher, MED-EL, Innsbruck, Austria). The eCAP was recorded consecutively from each CI contact, referenced to an external ground electrode (i.e., a contact located on the body of the implant package, outside the skull). Due to the known dependence of eCAP amplitude on the distance between stimulating and recording contacts (Gaertner et al., 2015; Takanen et al., 2024), we always chose the recording CI contact(s) adjacent to the stimulating contact. Whenever there were eCAP AGFs from both adjacent recording contacts available (i.e., not for the most apical and most basal contacts #1 and #12), we calculated the mean for each of the four analysed eCAP parameters.

**Figure 1.**
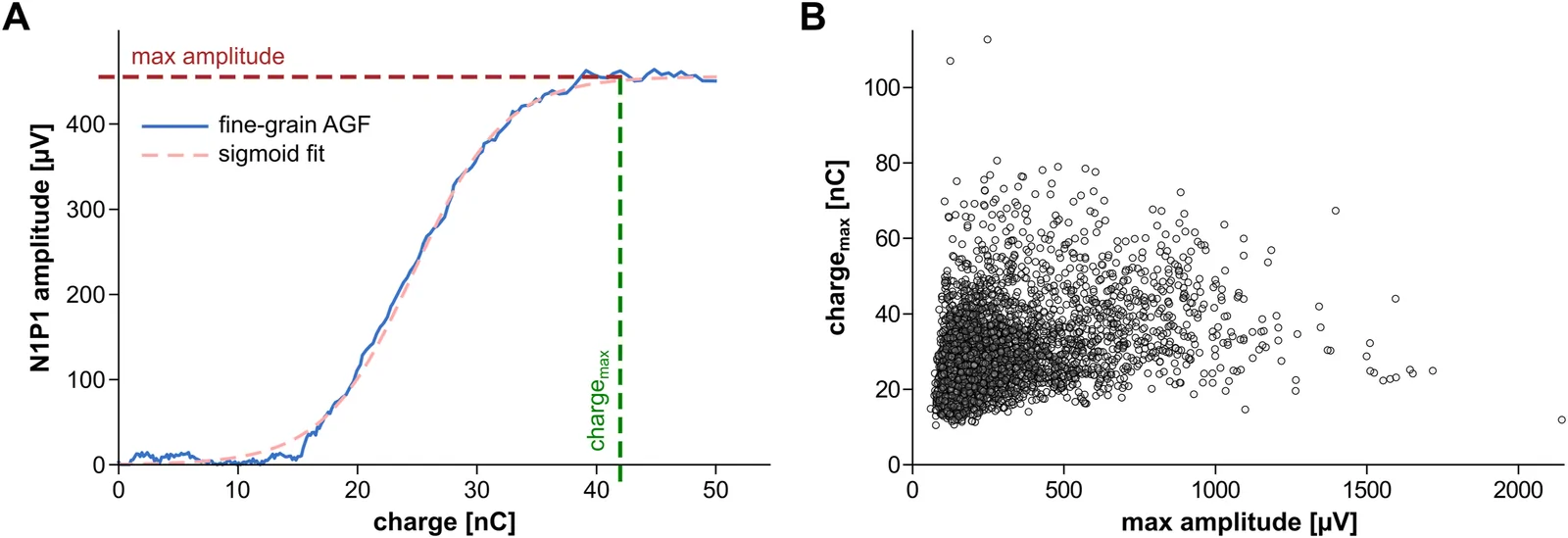
(A) Illustration of the procedure to derive the parameters required for FI calculation from the AutoART fine-grain amplitude growth function (AGF). Recorded data (blue) and the resulting sigmoidal function fitted to the AGF (red curve) are shown. The FI (here: 0.09 nC/µV) was calculated from the maximal N1P1 amplitude (red line) and the minimal charge level needed to reach saturation (charge_max;_ green line). (B) Distribution of all 4,082 corresponding maximal amplitudes and charge_max_-levels included in the study to calculate the FI

Analysis was restricted to AGFs that were scored as reliable by the internal criteria of the AutoART tool, as only these recordings provided all values required to describe the AGF function (including the threshold). Applying this quality criterion led to a drop out of 55% of AGFs (i.e., 16,648 from the initial 36,727), corresponding to a drop out of 27% of contacts for which recording from both adjacent contacts failed to reach the quality criterion and a drop out of 6% of ears for which no stimulation contact reached the quality criterion. The remaining AGFs were reliably fitted with the sigmoidal function (Figure 1A), resulting in goodness-of-fit of at least r^2^=0.974. As a sigmoidal function is symmetric around its inflection point (i.e., 50% of the dynamic range), the saturation response can be estimated accurately, as long as the inflection point has been captured. To confirm this point, we assessed potential differences in FI for saturating and non-saturating AGFs (see below).

Stimulation was delivered to each of the 12 contacts separately using biphasic single pulses of opposite leading-phase polarity for artefact reduction. The amplitude of the electric stimulation (in proprietary charge units, where 1 qu ≈ 1 nC) was steadily increased either until the user indicated that the sound was too loud, or until a pre-defined safe charge limit of each electrode contact (max 72 nC) was reached.

During the clinical settings, slightly different stimulation paradigms were used, with phase durations of 40, 50, or 60 µs and inter-phase gaps (IPG) of either 2.1 µs or 10 µs. Whereas changes in phase duration lead to differences in eCAP maximal amplitudes (Kruskal-Wallis test: H (2)=16.29; p=0.0003), this was not the case for the IPG (Mann-Whitney test: U=81387, p=0.7562). 

### Failure Index

The FI was calculated based on the AGF maximal amplitude (i.e., the upper asymptote of the sigmoid fit) and the minimum charge needed to reach saturation (i.e., charge_max_ at 99% of the upper asymptote; formula (1); Figure 1). We used charge rather than current to account for the phase duration effect on response amplitude (see above). This differs from the approach used in the animal study (Konerding et al., 2025). We found no significant effect of phase duration on the FI (Kruskal-Wallis test: H (2)=0.484; p=0.7853).

(1) $$FI=\frac{{charge}_{\max}}{Maxamplitude}\;\left[\frac{nC}{µV}\right]$$

We assessed the potential influence of non-saturating AGFs on the FI by analysing the FI as a function of the proportion of the dynamic range covered by the AGF. The dynamic range was calculated from threshold to charge_max_ (i.e., at 99% of the upper asymptote; see formula (1)) and the coverage was assessed based on the maximal stimulation charge reached during recording as per equation (2).

(2) $$coverage=\frac{\max\;stimulated-threshold}{{charge}_{\max}-threshold}*100\;\left[\%\right]$$

From our previous results (Aljehani et al., 2025), we postulated that 70% coverage would be enough to achieve reliable estimates of the FI (as per equation (1)). Therefore, we binned the coverage into three categories: <70%, 70–100% and >100%.

### Correlation With Speech Scores

The most frequently used speech recognition test in our cohort was the Freiburger monosyllabic word test (Hahlbrock, 1953; Hochmair-Desoyer et al., 1997) performed in quiet. Additionally, we included scores on the German Hochmair-Schulz-Moser (HSM) sentence test both in quiet and in noise. The HSM test consists of 30 lists of 20 everyday sentences, with each list consisting of 106 words (Hochmair-Desoyer et al., 1997). Scores were defined as the percentage of correctly repeated words. For the HSM test in noise, speech-shaped noise was presented at a signal-to-noise ratio of -10 dB. The acoustic stimuli were presented from a single loudspeaker. The users listened via their everyday audio processor strategy. In cases of single-sided deafness, the second ear was occluded or acoustically masked during the test. The eCAP parameters were tested for correlation with the maximal (best) speech scores during the investigation period to reduce the influence of performance limiting effects during the clinical assessments (M3–Y1; see discussion).

For this analysis, we introduced inclusion and exclusion criteria common to the field: we included one ear per native speaking CI user with postlingual hearing loss (Kludt et al., 2025; Scheperle & Abbas, 2015; Schvartz-Leyzac & Pfingst, 2018) and we excluded users with substantial residual acoustic hearing in the tested ear (Dawson et al., 2025; Kludt et al., 2025). We further excluded five users with comorbidities that could affect speech understanding (i.e., tinnitus, developmental disability, and ‘difficulty finding words’). The resulting sample sizes differed based on the speech perception test used (since not all tests were carried out with all users). The sample sizes for each comparison are indicated in the results section.

Due to collinearity between the different CI contacts (variance inflation factor, VIF >5), it was not possible to combine the measures from all contacts in one linear model to predict speech understanding scores. Therefore, we assessed two alternative approaches: 1) using the mean FI value per ear (irrespective of the number of contacts included) and 2) using the maximum (worst) FI value per ear, with additional exclusion criteria for optimized outcomes (below). The maximal FI was chosen (analogous to the guinea pig study; Konerding et al., 2025), as we posit that foci of reduced neural health are more detrimental to speech understanding than a homogeneous reduction in neural health across all contacts (DeVries et al., 2016; Pfingst et al., 2004; Zhou & Pfingst, 2014). The choice of using extreme values along the array is linked to the proposed clinical application of deactivating the “worst” CI contact to improve speech understanding.

Ideally, extremes should only be assessed when FI values are available for all 12 CI contacts. As this restriction would lead to further reductions in sample size, we chose those ears for which the FI could be determined for a uniform set of ten contacts (i.e., contact #1 through #10). We excluded basal contacts from this set because these have been found to be less important for speech understanding (Shannon, 2002; Zamaninezhad et al., 2023). All ears had full insertions, which is also a recommended inclusion criterion (e.g., Skidmore et al., 2023).

We assessed whether the resulting linear equation for the correlation with the highest coefficient of determination was similar to the one observed using the subset of ears with all n=12 contacts included (n=20; see results).

According to personal communication with the audiologists conducting the tests, the rapid sequence of sentences in the HSM task could result in a withdrawal of some patients, leading to the tests being pre-terminated. In this second analysis, we therefore decided to include only non-zero HSM scores (i.e., with at least 1 word correct over the whole testing period; the reduced samples sizes are indicated in the results section). This was to increase likelihood that the speech scores would be informative of neural health (as opposed to cognitive factors).

To put the results with the FI into perspective, we chose two comparisons: 1) we compared the correlation coefficients with the FI to those resulting from other eCAP parameters (i.e., threshold, slope and maximal amplitude) and 2) we included two conventional factors: age at implantation (which corresponds to age at testing) and duration of hearing loss (as per self-report). In the second approach, we compared two multiple linear regressions to predict speech outcomes; one without FI, and one for which we added the FI as additional factor.

### Correlation With Electrode-to-Modiolus Distance (EMD)

**Figure 2.**
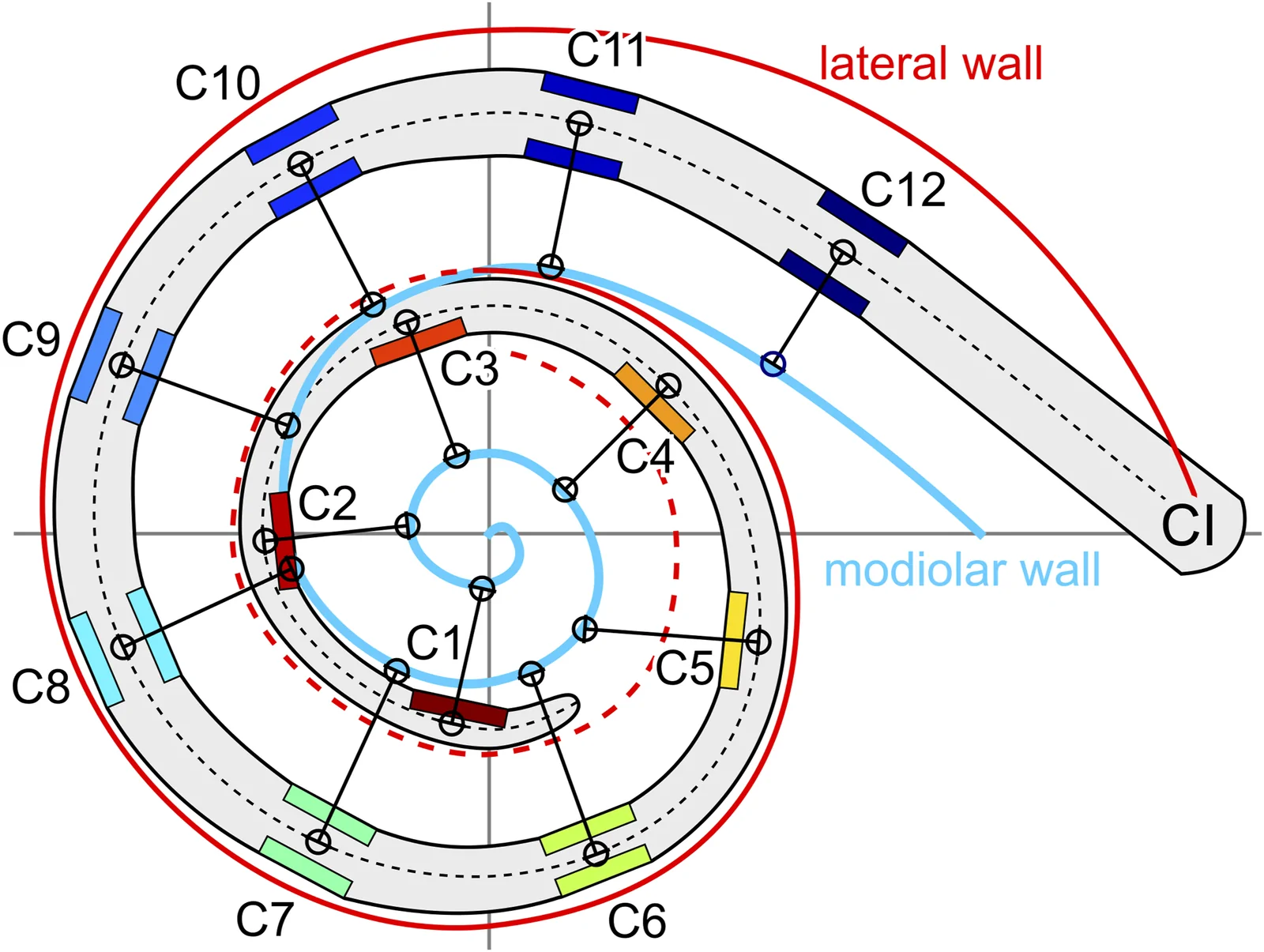
Assessment of the electrode-to-modiolar wall distance (EMD) from 3D cochlear reconstructions. The reconstructed scala tympani is shown, overlaid by the reconstructed CI array from apical contact C1 to basal contact C12. The EMD (black, connecting line) was measured from the midpoint of the array at the position of each contact to the edge of the scala tympani. The modiolar wall (blue) was reconstructed based on the scala width from the lateral wall (red; dashed line indicates the progression of the scala wall covered by more apical turns)

To approximate the distance of the CI contacts to the excitable structures, we calculated the EMD from the centre of the electrode contact to the modiolar wall (Garcia & Carlyon, 2025; Salcher et al., 2021) in 227 ears and a total of 1,907 contact positions. 3D cochlear reconstructions were computed based on cone-beam computed tomography imaging (Xoran XCAT or the Morita ENT devices). This imaging was performed during clinical routine at the Hannover Medical School, as previously described (Salcher et al., 2021; Timm et al., 2018). Segmentation of the lateral cochlear wall was performed using the pre-operative image (Meng et al., 2016; Schurzig et al., 2018b; Würfel et al., 2014). Segmentation of the CI contact positions was performed with the post-surgical image. Both operations were performed using the software tool OsiriXMD (version 2.5.1 64bit, Pixmeo SARL, Switzerland). These segmentations were registered as described in Schurzig et al., 2018a, and the shape of both the inserted electrode array as well as the scala tympani were reconstructed using the same methodology as in the recently proposed virtual implantation (Schurzig et al., 2023), yielding a geometric representation from which the EMD of the individual contacts was estimated (Figure 2). As with the correlations with perceptual outcomes, we compared the dependence of FI, threshold, slope and the maximal amplitude on EMD.

### Statistics

Most statistical tests were performed using GraphPad Prism 11 (GraphPad Software, Inc., Boston, MA, USA). We used the Bonferroni correction in the post-test comparisons to correct for multiple testing. The Kolmogorov-Smirnov test was used to test the assumption of normality. Where this assumption was violated, we used non-parametric tests. To assess correlations, we used the Pearson or Spearman method where appropriate. Additionally, we report the linear regression coefficients (e.g., correlation with speech scores). To assess non-linear relations, we used regression curves and reported correlation coefficients for significant correlations.

Additional multilevel analyses were performed in JASP (JASP Team (2025), Version 0.17.1) and GraphPad Prism 11. Repeated measure ANOVA was used to assess the effects of EMD and contact (fixed factors), with ear as random factor. Multiple linear regression was used to assess the combined effects of age at implantation, duration of hearing loss and FI on performance in the speech-in-noise test. Two models, one without FI (HSM noise ∼ Intercept + age + duration) and one with FI (HSM noise ∼ Intercept + FI + age + duration) were compared via AIC and r^2^-values. Missing values for duration of hearing loss (n=7) were interpolated by the model.

## Results

As there were no significant differences in average FI between males and females (Mann Whitney: U=8603, p=0.1699), left and right ears (Mann Whitney: U=9188, p=0.5491), and whether or not the CI was used with an additional acoustic component at the tested ear (Mann Whitney: U=2212, p=0.9839), we combined all ears for further analyses, selecting sub-groups of the dataset according to the particular analysis (detailed below and in the methods section).

### Stability Over Time

**Figure 3.**
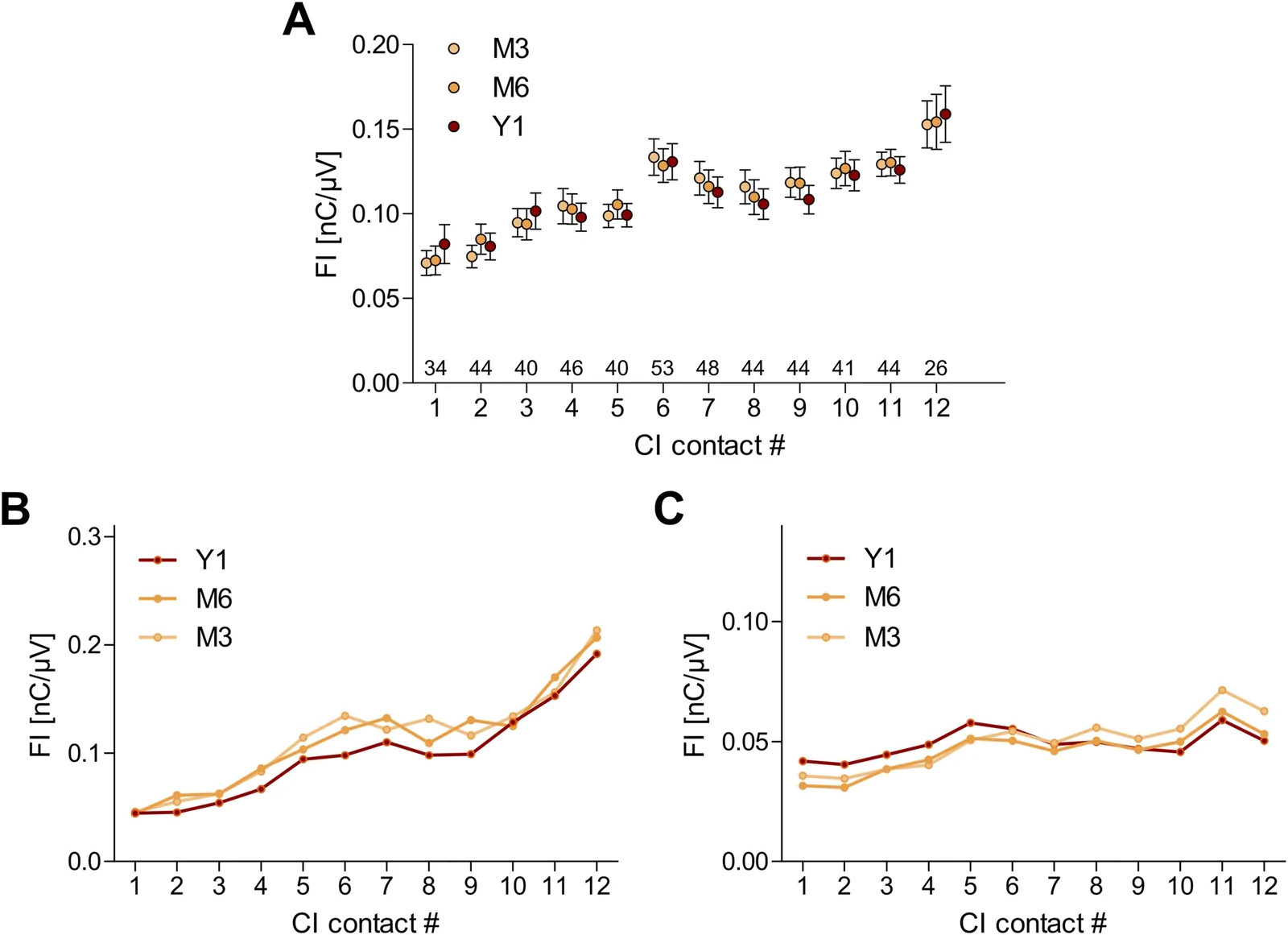
(A) Stability of FI values over time, from 3 months (M3), to 6 months (M6), and 1 year (Y1) post-surgery. Given are mean values with the standard error of the mean (SEM). Number of ears per contact are given above the x-axis. (B and C) Two representative ears (selected from panel A) with low mean FI values (B: 0.05 nC/µV, C: 0.10–0.11 nC/µV) and distinct, but stable patterns along the array. Note the difference in axis scaling

To take into account the individual differences between contacts, we assessed the influence of recording time (i.e., 3, 6, and 12 months post-surgery) on FI using two-way ANOVA, combining contacts and time. This analysis was performed for a subset of ears which were tested at all 3 time points (n=70, Figure 3A). Whereas there was an overall significant difference between contacts (details below), there was no significant difference based on recording time from M3 until Y1 post-surgery, and also no interaction between recording time and contact (Contact: F (11, 1476)=16.08, p<0.0001; Time: F (2, 1476)=0.0648, p=0.9373; Interaction: F (22, 1476)=0.1972, p>0.9999; Figure 3). Due to the stability over time, we averaged the FI over the analysis period (M3–Y1), which increased overall statistical power, as not all ears were tested at every time point.

### Robustness to Non-Saturating AGFs

**Figure 4.**
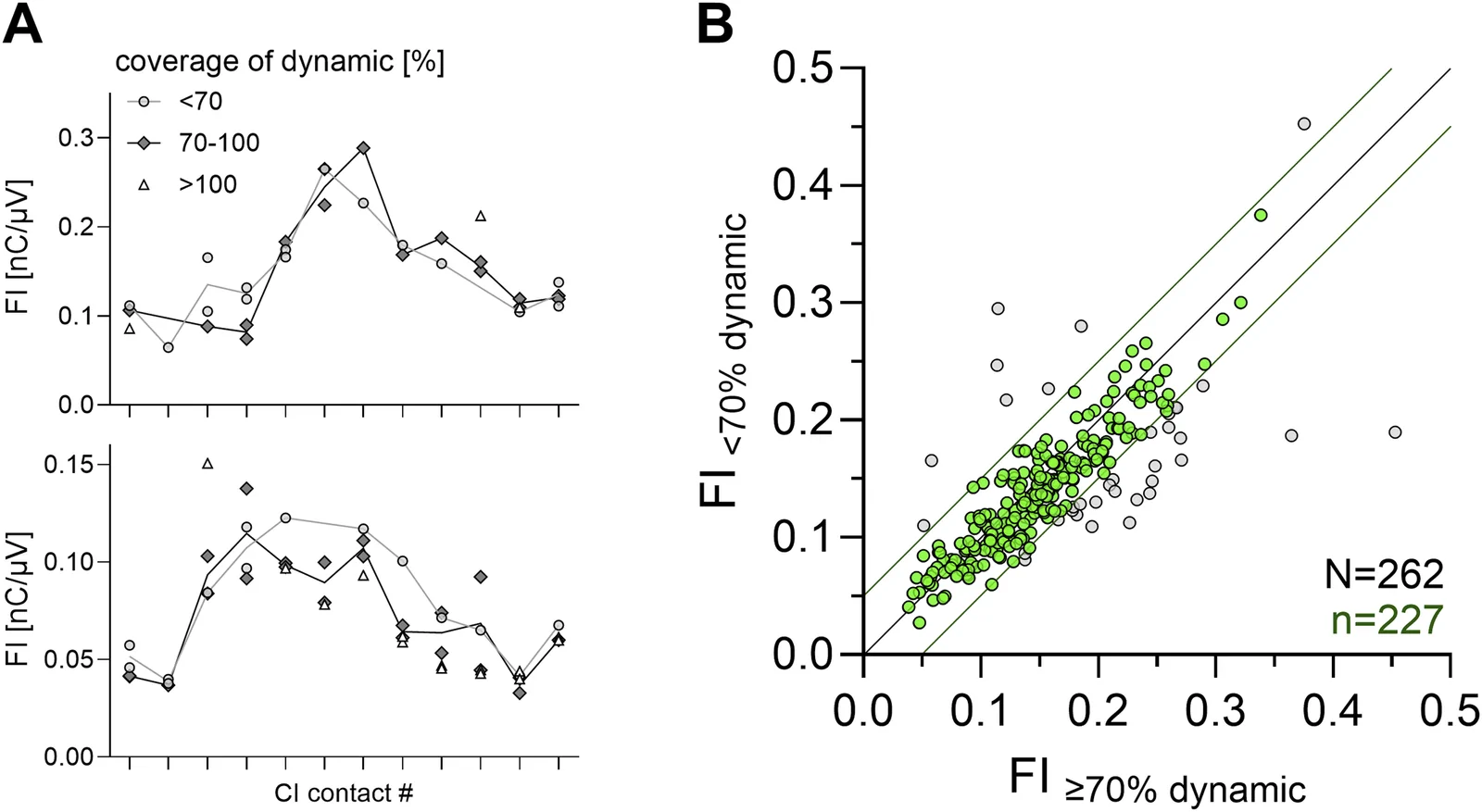
Similarity of the FI calculated from AGFs with varying dynamic range coverage. (A) FI values are given for each contact of two ears, with either overall high FI (top) or low FI (bottom). FI values from all eCAP test sessions conducted throughout the test period (month 3 till year 1 post-surgery) are indicated (symbols) and coloured depending on how much dynamic range was available for the AGF (light grey: less than 70% of the dynamic, dark grey: 70–100%, white: into saturation). The medians are connected for the coverage categories <70% (grey line) and 70–100% (black line) showing good correspondence throughout the array. (B) The average FI values for each ear, separately calculated from AGFs with at least 70% dynamic range coverage and those with less, were highly correlated. The data was scattered around the identity line with most data deviating not more than ± 0.05 nC/µV from it (green)

From a total of 37,641 recordings, 63.4% of the AGFs covered at least 70% of the dynamic range, of which 7.1% were saturating AGFs (i.e., >100% coverage). In two cases for which we had recordings of all three coverage categories throughout the array, we demonstrated that the FI was similar across all contacts (Figure 4A). In 91 ears for which we had recordings from all three coverage categories, we assessed paired differences between average FI of the three coverage-categories and found no significant difference (repeated measure ANOVA: F (1.387, 124.8)=1.696, p=0.1949). To include more data for assessing paired associations, we combined the two groups with coverage of at least 70% of the dynamic range (≥70%). The average FI per ear, based on <70% coverage and ≥70% coverage, were found to be highly correlated (Spearman rho=0.835, p<0.0001, n=262), with 86.6% of the ears scattered ± 0.05 nC/µV around the identity line (Figure 4B).

### Dependence on Age

**Figure 5.**
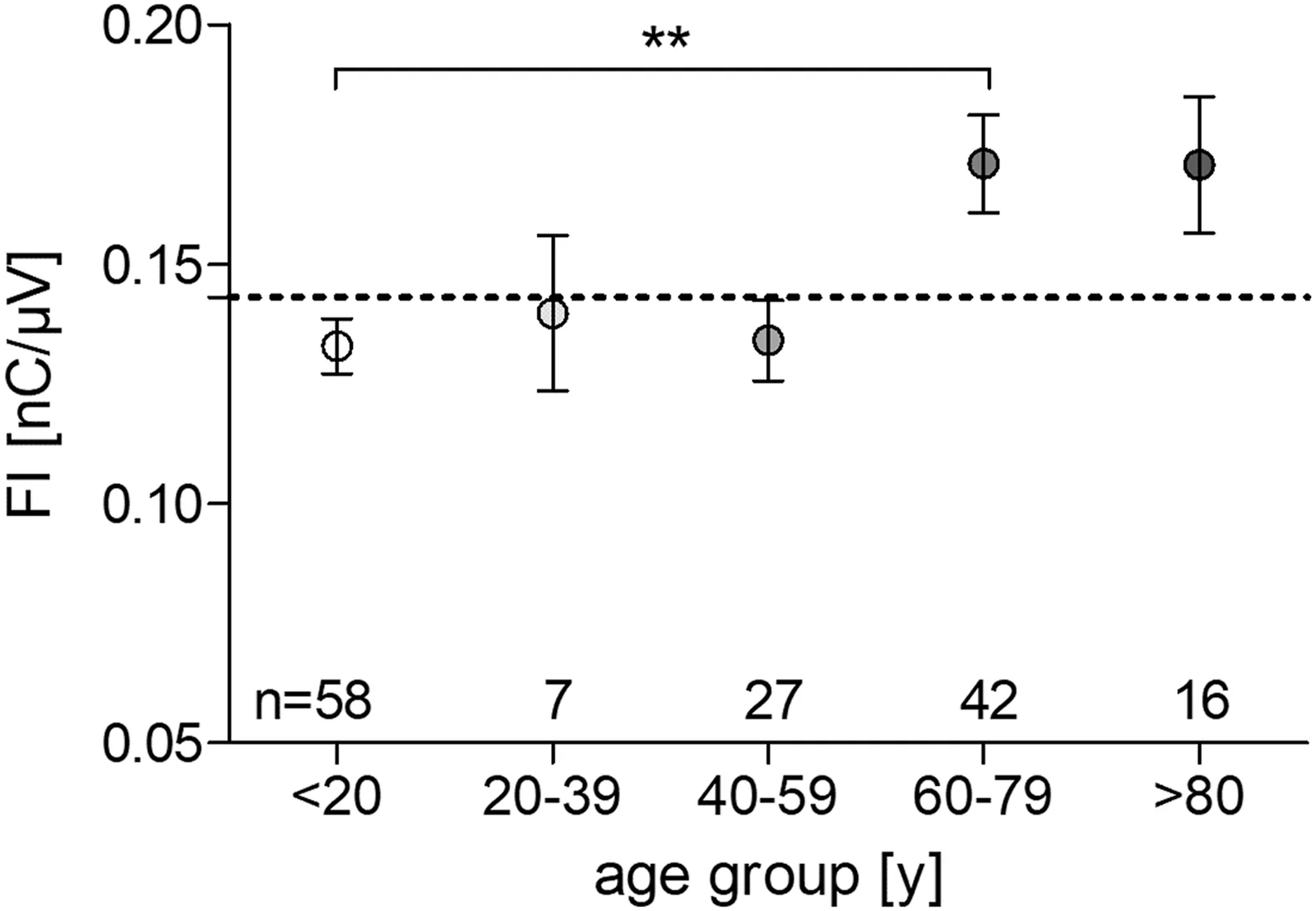
Increase of mean FI (M3–Y1) with age for combined ears with pre- and postlingual hearing loss. Given are means (symbols) and SEM (whiskers) for each age group (bin-size: 20 years). Sample sizes per group are indicated above the x-axis. The dashed line indicates the grand median FI_GM_=0.143 nC/µV for the median split approach

We analysed the correlation of the mean FI per ear with age at implantation. As the causes of hearing loss differed between ears with pre- and postlingual hearing loss (details below), we restricted the analysis to cases with idiopathic etiologies. There was a significant increase of FI with age for the combined ears with pre- and postlingual hearing loss (Spearman: rho=0.220, p=0.0070, n=150). The regression yielded higher coefficients of determination for non-linear (i.e., sigmoid: r^2^=0.132) than linear regression (r^2^=0.072), indicating a rapid rather than gradual change in FI with a significant increase in FI for the group 60–79 years relative to the youngest age group (1-way ANOVA: F (4, 145)=4.478, p=0.0019 with Bonferroni corrected post-test; Figure 5).

### Influence of Etiology

For most ears, the etiology was idiopathic (109 post-lingual, 57 pre-lingual). The majority of known etiologies were unique to either post- or pre-lingual cases. The most common etiology was sudden hearing loss (n=45, only post-lingual), followed by genetic etiologies (6 post-lingual, 8 pre-lingual), Menière’s disease (n=12, only post-lingual), auditory nerve hypoplasia (n=7, only pre-lingual), and trauma (n=6, only post-lingual). Due to these differences in the distributions of etiologies, as well as the association between FI and age, we assessed the influence of etiology on FI separately for ears with post- and prelingual hearing loss.

**Figure 6.**
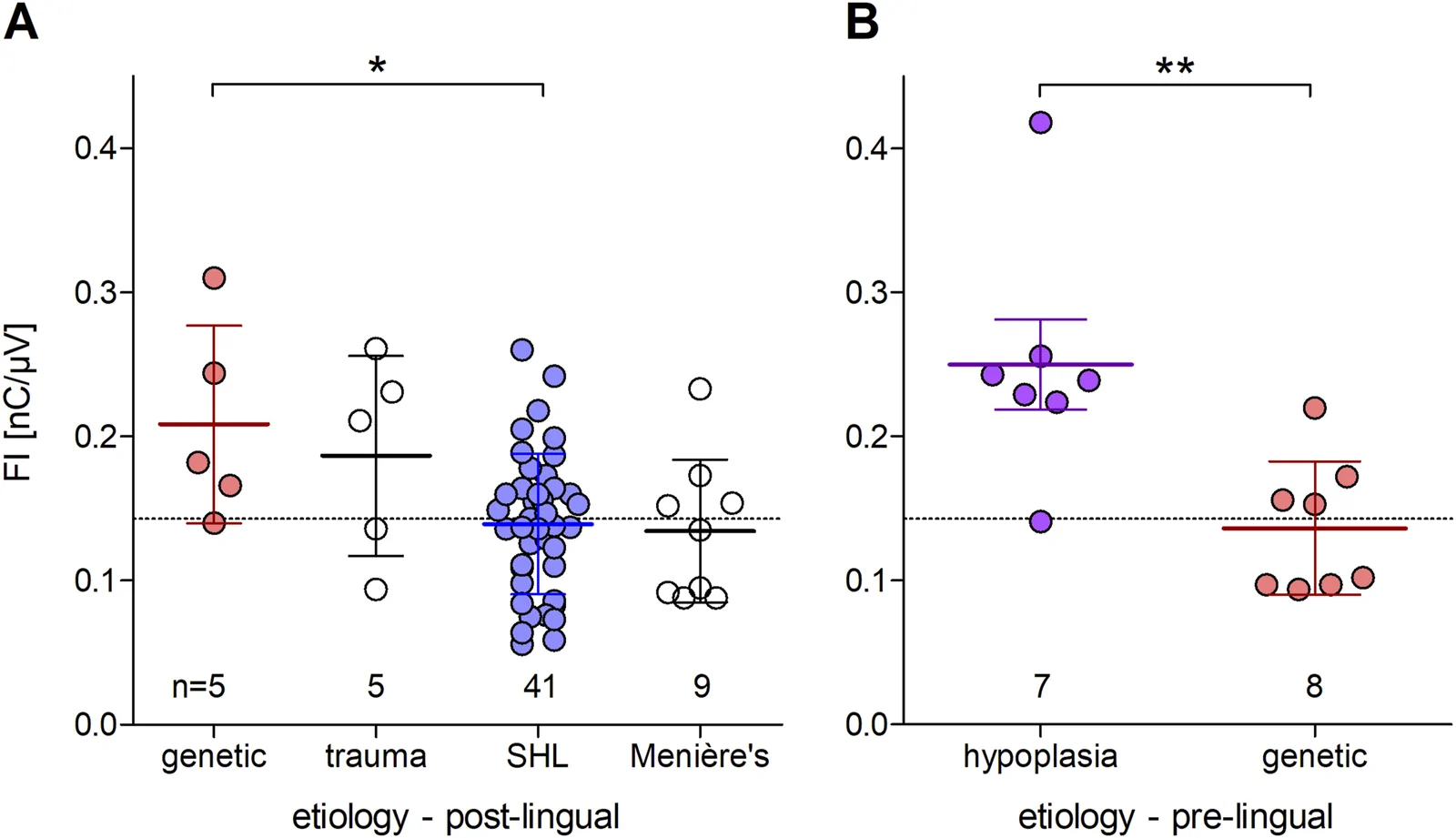
Differences in mean FI (M3–Y1) based on the most common etiologies within the study’s database for ears with (A) postlingual and (B) prelingual hearing loss. Given are group means (lines) and SD (whiskers), overlaying single cases (symbols). Sample sizes per group are indicated above the x-axis. The dashed line indicates the grand median FI_GM_=0.143 nC/µV for the median split approach. SHL – sudden hearing loss

In the postlingual hearing loss group, there was a significant difference in FI based on etiology (one-way ANOVA: F (3, 56)=3.720, p=0.0164; sample sizes given in Figure 6) with hearing loss of unspecified genetic origin associated with significantly higher FI compared to sudden hearing loss. In the prelingual hearing loss group, individuals with cochlear nerve hypoplasia had significantly higher FI compared to those with unspecified genetic etiologies (Mann Whitney: U=4.000, p=0.0065, Figure 6B). The difference in FI between the post- and the prelingual ears with genetic etiologies (Figure 6A and B – red) may be attributed to the age difference between these two subgroups: post-lingual (35–76 years) and pre-lingual (1–22 years). Concordantly, the FI significantly increased with age at implantation for the 13 ears with congenital hearing loss (Pearson r=0.658, p=0.0146; linear regression: r^2^=0.433).

### Dependence on Stimulation Contact

Based on our findings in the animal model (Konerding et al., 2025), we hypothesised that the FI indicated the neural health profile, reflecting variability of SGN integrity along the cochlea. Therefore, we selected ears with high average FI (i.e.; FI_M3–Y1_>0.143; see Median split approach) for which at least 11 contacts were available.

**Figure 7.**
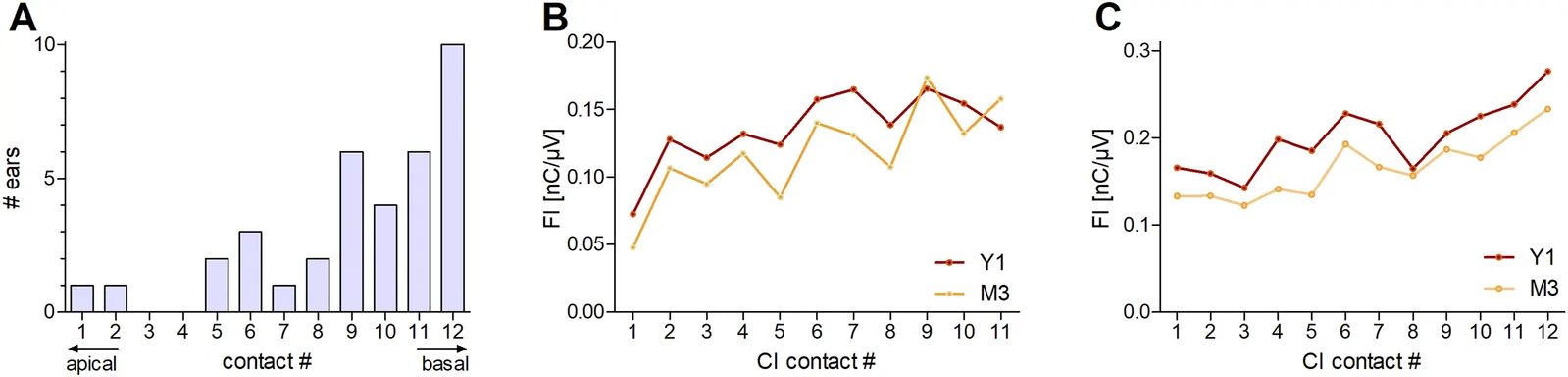
(A) Histogram of the position of FI maxima along the array from contact 1 (most apical) to contact 12 (most basal) for ears with overall high FI (i.e., mean FI> 0.143; N=36). (B and C) Two representative ears with high mean FI at Y1 (A: 0.14, B: 0.20) show distinct and reproducible FI pattern with multiple peaks and troughs along the array at three months (M3) and one year (Y1) post-surgery. Note the difference in scaling for (B and C)

The maxima of these 36 ears showed a wide variability of positions from contact #1 to contact #12, with most cases peaking at basal contact #12 (n=10), followed by contacts #9 and #11 (n=6, each; Figure 7A). In two representative cases for which data was available at both M3 and Y1 (Figure 7B and C), the overall stability of the peaks and troughs in FI pattern along the array was confirmed.

### Correlation With Speech Perception

As a first step, we correlated mean eCAP parameters with speech perception scores (non-parametric Spearman correlation; Table 1), using a large dataset (inclusion criteria: 1^st^ ear or unilaterally implanted German speakers with postlingual hearing loss, without acoustic hearing in the tested ear) without restriction due to number of CI contacts or potential floor effects in speech perception.

**Table 1.** Results of Spearman Correlation Between Mean eCAP Parameters (FI, Threshold, Slope and Maximal Amplitude) and Percent Correct Scores on Three Speech Perception Tasks. Shown are the Spearman Rho Coefficients and p-Values. Significant Correlations are Indicated in Bold

Spearman	Mean FI	Mean threshold	Mean slope	Mean amplitude
Monosyllabic (n=181)				
rho	**-0.197**	0.006	**0.184**	**0.193**
p	**0.0079**	0.9363	**0.0130**	**0.0093**
HSM quiet (n=143)				
rho	-0.153	0.0384	**0.168**	0.119
p	0.0686	0.6486	**0.0453**	0.1566
HSM noise (n=145)				
rho	**-0.271**	0.082	**0.260**	**0.252**
p	**0.0010**	0.3247	**0.0016**	**0.0023**

There was no significant correlation between the threshold with any speech test. The correlations were similar for FI, slope and maximal amplitude, with a significant relationship between the eCAP parameters and speech perception in the monosyllabic test (very weak: r<0.2) and the HSM in noise (weak: r∼0.3).

In a second step, we correlated speech perception scores with the extreme values of the putative markers (maxima of FI and threshold; minima of slope and amplitude). For these extremes to be representative for the respective ears, it was necessary to restrict the analysis to those patients for which a uniform set of ten contacts were included in the analysis.

**Figure 8.**
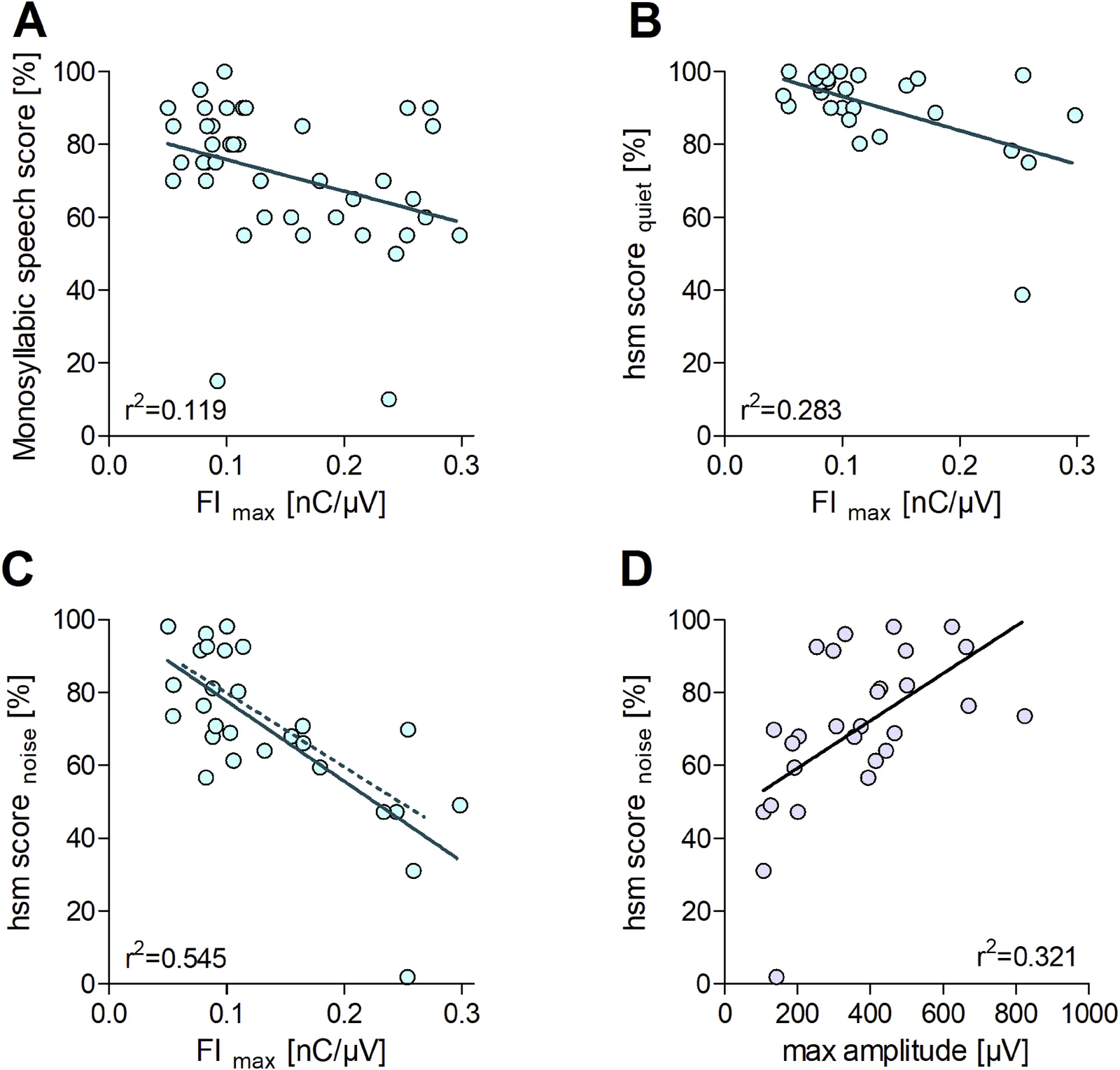
Correlation of eCAP parameters with speech test outcomes for ears for which the same n=10 contacts were available to calculate the extreme values along an array (i.e., maximal speech score, maximal FI, and minimal value of the maximal eCAP amplitude; all for M3–Y1). The FI was significantly correlated with (A) monosyllabic speech scores, (B) HSM scores in quiet, and (C) HSM scores in noise. (D) The maximal eCAP amplitude significantly correlated with HSM scores in noise. (A–D) Given are the linear regression line (solid line) and the respective coefficient of determination (r^2^). (C) The additional linear regression line (dashed line) indicates the dependency of FI on HSM score in noise when the maximal FI was calculated based on a subset of ears for which all 12 contacts were available

The results were similar to the previous analysis in that all assessed eCAP parameters, except the threshold, showed significant relations to speech outcomes (using Pearson correlation due to normal distribution). However, the strength of the correlation was higher than for the large population, reaching moderate levels (r∼0.4) for the remaining three eCAP parameters (i.e., FI, slope, and maximal amplitude) and all tests. The correlation coefficients increased from monosyllabic word test, over HSM in quiet to HSM in noise. For both sentence tests (quiet and noise), the FI reached the highest correlation coefficients (i.e., in absolute terms) of all tested eCAP parameters (Figure 8, Table 2).

**Table 2.** Pearson Correlation Coefficients Between Extreme (Maximum or Minimum) eCAP Parameters (FI, Threshold, Slope and Maximal Amplitude) and Percent Correct Scores on Three Speech Perception Tasks. Given are Pearson r and p-Values, With Significant Correlations Indicated in Bold

Pearson	Max FI	Max threshold	Min slope	Min amplitude
Monosyllabic (n=41)				
r	**-0.349**	0.072	0.281	**0.405**
p	**0.0273**	0.6838	0.0750	**0.0087**
HSM quiet (n=27)				
r	**-0.532**	-0.017	**0.415**	**0.403**
p	**0.0043**	0.9477	**0.0312**	**0.0374**
HSM noise (n=28)				
r	**-0.738**	-0.253	**0.605**	**0.567**
p	**<0.0001**	0.3107	**0.0006**	**0.0017**
Linear r^2^	**0.545**	0.064	**0.366**	**0.321**

Based on the coefficients of determination (r^2^) the variance in maximal amplitude explained 32% of the variance in speech outcome (Figure 8D), the variance in slope explained 37%, and the variance in FI explained more than half of it (Figure 8C). For the FI, we confirmed that the regression line derived for this reduced data set (10 contacts: y = -220*x + 100; Figure 8C solid line) was similar to one for which all 12 contacts were available (resulting in an even smaller subset of ears; n=20: y = -203*x + 100; Figure 8C dashed line).

In a last step, we assessed the contribution of the FI to speech understanding in relation to two conventional predictive factors: age and duration of hearing loss. We calculated two models, one with FI and one without it.

The first model (age+duration) indicated that the two predictors explained 30% of the variance in speech perception (r^2^= 0.3013, F (2, 18)= 3.880, p=0.0397, AIC= 135.8). Only age significantly predicted speech understanding (ß= -0.8543, p= 0.0130), whereas duration of hearing loss did not (ß= -0.3152, p= 0.5139).

By including the maximal FI as third main effect, the model improved (i.e., decreased AIC= 123.5) with the three predictors explaining 67% of the variance in speech perception (r^2^= 0.6698, F (3, 17)= 11.50, p=0.0002). In this model, only FI significantly predicted speech understanding (ß= -232.9, p=0.0004), whereas both age and duration of hearing loss did not (age: ß= -0.2713, p= 0.3058, duration: ß= -0.4174, p= 0.2305).

### Independence of FI on EMD

**Figure 9.**
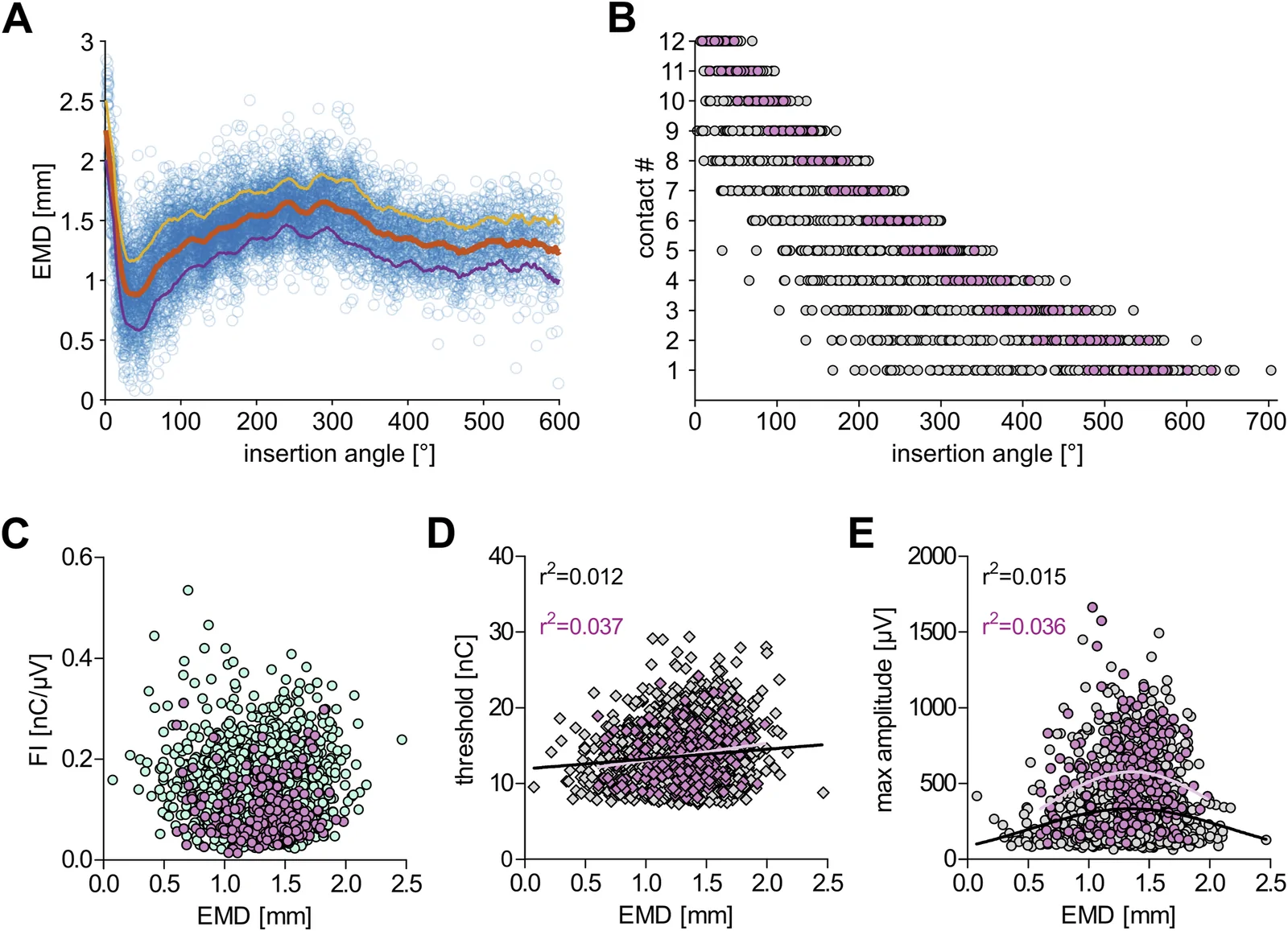
(A) The electrode-to-modiolar wall distance (EMD) changed non-monotonically with insertion angle. (B) The contacts in the full data set (grey symbols, N=1,907) covered a wide range of insertion angles per contact number. This spread was substantially reduced for a subset of data with full insertions (pink symbols, N=247; selection criteria as per speech correlation; Fig. 8C). (C–E) The dependence of the different eCAP parameters on EMD differed according to our hypotheses: (C) no correlation with FI, (D) correlation with eCAP threshold and (E) with maximal eCAP amplitude. (D–E) The linear (threshold) and non-linear regressions (max amplitude) and the resulting coefficients of determination are given for both the full (black) and the reduced data set (pink)

We assessed the dependence of eCAP parameters on the distance to excitable structures by correlating the parameters (as a group) with the EMD. For this, we used all available data including partially inserted CIs (N=1907 contacts; Figure 9A and B: grey symbols). We repeated the analysis with the subset of ears (n=28) that was used for speech-in-noise correlation (i.e., all full insertions; N=247 contacts; Figure 9B: pink symbols).

EMD changed non-monotonically with insertion angle, decreasing rapidly within the first 50° beyond the round window, then reaching a maximum at around 300° (Figure 9A). Due to differences in morphology and insertion depth, the contact number (#1–12) did not predict the insertion angle (Figure 9B). Thus, each contact was included independently in the correlation analysis.

FI was not significantly associated with EMD, either for the full data set (Spearman rho=-0.025, p=0.2666, Figure 9C) or the reduced data set (Spearman rho=-0.026, p=0.687). The eCAP threshold was significantly affected by distance, with a linear increase in thresholds with increasing distance from the modiolar wall both for the full data set (rho=0.088, p=0.0001; Figure 9D) and the reduced data set (rho=0.198, p=0.0018). The maximal eCAP amplitude showed an intermediate dependence on EMD, which resulted in a significant correlation in the full data set, with highest amplitudes for intermediate EMDs (rho=0.055, p=0.0163; gaussian fit; Figure 9E) and a lack of significant association in the reduced data set (rho=0.110, p=0.0860). The slope was not dependent on the EMD (full data: rho=0.037, p=0.1082; reduced: rho=0.020, p=0.7584).

Due to the potential influence of contact (fixed effect) and ear (random effect) on the data, we complemented the regression analysis with a repeated measure ANOVA. We confirmed that there was no significant effect of EMD on the FI (EMD F (1, 224.08)=1.143, p=0.286, contact F (11, 1657.37)=3.626, p=<0.001, EMD*contact F (11, 1654.69)=1.289, p=0.224). Both the threshold and the maximal amplitude show a more complicated dependence on EMD, with a significant interaction between EMD and contact, reflecting the average change in EMD with contact position in the apical-basal dimension (threshold: EMD F (1, 238.14)=36.389, p<0.001, contact F (11, 1503.36)=1.932, p=0.032, EMD*contact F (11, 1641.85)=4.232, p=<0.001; amplitude: EMD F (1, 254.74)=5.513, p=0.020, contact F (11, 1346.91)=6.877, p<0.001, EMD*contact F (11, 1442.89)=2.362, p=0.007).

### Median Split Approach for Dichotomization

Based on the results in the animal model, and in the absence of histological confirmation from temporal bones, we suggested that a dichotomization of the FI using the group median (i.e., median-split approach; DeCoster et al., 2011) could be used to stratify the patient population to identify those ears that have substantial reduction in neural health (i.e., average FI > FI_GM_). The median FI over all 199 ears with postlingual hearing loss was 0.147 nC/µV and the median over all 79 ear with prelingual hearing loss was 0.136 nC/µV. There was no significant difference between ears with pre- and post-lingual hearing loss in average FI (Mann-Whitney: U=7346; p=0,3952). As such we combined them as FI_GM_=0.143 nC/µV.

The FI_GM_ was indicated in the results on age (Figure 5, dashed line) and etiology (Figure 6, dashed line) and was in line with the conventional analyses using continuous FI values. The FI was below FI_GM_ for the age groups less than 60 years (average FI <0.143) and above it in the age groups greater than 60 years. Significant changes in FI based on etiology were observed: The auditory nerve hypoplasia group was significantly elevated above the FI_GM_ (Wilcoxon test vs FI_GM_: W=26, p=0.0313), with the lowest value (FI=0.141nC/µV) being close to FI_GM_.

We further assessed potential effects of AGF coverage on the grand-median split approach. When the FI_GM_ was calculated solely based on data derived from AGF with >100% coverage it slightly decreased to 0.138 nC/µV. This change did not affect the above conclusions. As can already be inferred from Figure 4C, some ears changed their allocation based on the FI_GM_: Of 172 ears available for the comparison, 83.1 % kept their assignment as either high (>FI_GM_) or low (<FI_GM_) irrespective of AGF coverage. Of the 16.9% changing their assignment, 10 ears (5.8%) changed from high to low FI with reduced coverage (i.e., <70%) and 19 ears (11.0%) changed from low to high FI with reduced coverage.

### The Efficiency Index

In a recent publication, the inverse of the FI (the Efficiency index) was introduced as potential alternative measure (i.e., Efficiency index; Meuser et al., 2026).

**Figure 10.**
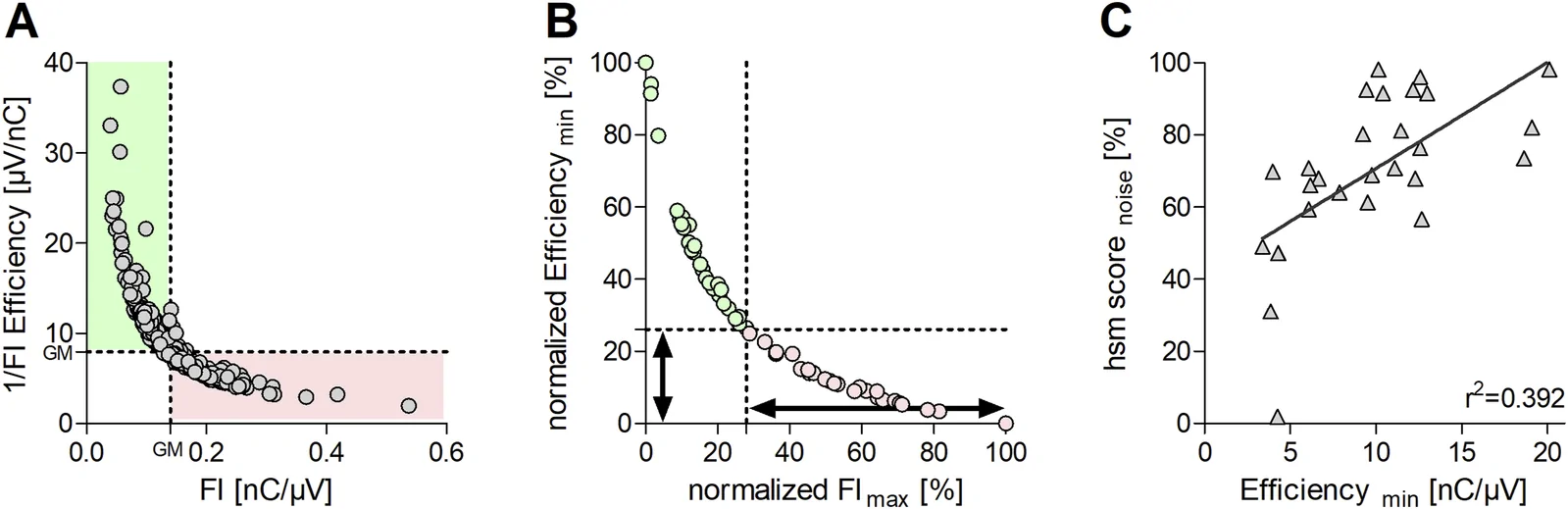
(A) The mean FI and the mean Efficiency Index for each ear (N=278) usually, but not always, led to similar conclusions based on the median split approach: a high number of ears fell on the same side of the grand median (GM) for both measures (“better” neural health: green shading; “worse” neural health: pink shading). (B) The min-max normalisation of extreme values along the array (n=55) highlights the main difference between the two measures: the ears suggested to have aggravated reduction of neural health (pink symbols) cover more than 70% along the FI dimension (vertical arrow), but less than 30% along the Efficiency dimension (horizontal arrow). Correspondingly, (C) the variance in Efficiency explains less than 40% of the variance in speech in noise (same n=28 ear as in Figure 8C and D)

When applying the median split approach, the two measures lead to differences in stratification: the classification differed between the Efficiency Index and the FI in 26 cases (9%; Figure 10A). Thereby, the range of values for ears falling on the side of the FI, suggested to indicate better neural health (Figure 10 A&B: green) and ears falling on the side with presumed reduced neural health (Figure 10A and B: pink) differed between the two measures. Whereas the largest portion along the Efficiency axis corresponded to ‘better health’, the largest portion along the FI axis corresponded to ‘reduced health’ (∼70%; Figure 10B).

This difference in separation precision led to differences in the predictive value for speech outcomes: While the variance in Efficiency index was significantly associated with speech understanding, the correlation coefficients were lower than those for the FI (Pearson correlation: monosyllabic r=0.348, p=0.0257, HSM quiet r=0.436, p=0.0229, HSM noise r=0.627, p=0.0004; compare with Table 2). The Efficiency Index explained about 40% of the variance in speech-in-noise test scores (r^2^=0.393, Figure 10C), whereas the variance in FI explained nearly 55% of the variance in these scores.

## Discussion

Here we provided evidence consistent with the hypothesis that the FI is a suitable marker of the neural health profile according to five criteria: 1) stability over several months post-surgery, 2) group differences, 3) correlation with outcomes, 4) individual profiles along the array, and 5) independence from electrode-to-modiolus distance.

### Limitations

This study had two main limitations that are general to retrospective studies using post-surgical clinical eCAP recordings: we had an uncontrolled research sample and we needed to include quality criteria that may induce selection bias. Due to the clinical origin of the data, several factors were not controlled for such as demographics (e.g., sex, age, and hearing status), stimulation paradigms (e.g., different phase durations and IPGs), and performance assessment (e.g., not all tests in all patients). We therefore assessed the different, potentially influencing factors and discuss significant findings below.

Similar to a cohort study of a different CI manufacturer (Advanced Bionics, Larsen et al., 2026), we did not find any significant influence of sex or implant side, and we pooled the data accordingly. The necessity to restrict analyses to recordings of sufficient quality (Strahl, S. et al., 2018) may have led to biased sample sizes, as contacts with weak and less reliable eCAP responses were more likely to be excluded. A recent cohort study (Larsen et al., 2026) with similar numbers of AGFs as reported here (45,912 versus 36,727), retained only 27% of the initial number after applying quality criteria. This retention rate was substantially lower than the 45% retained in the present study. Furthermore, drop-out was distributed throughout our research sample, with only 6% of ears being removed from the sample due to the quality criterion, making it unlikely that whole sub-groups (e.g., poor performers) were removed from the data set. Further selection criteria oriented towards studies utilizing similar methodologies to form more uniform sub-populations (e.g., correlation with speech scores). Although this further reduced the sample size, it increased comparability to other studies (detailed below).

Another limitation to all studies using human eCAP recordings is that recordings often have to be terminated before the AGF reaches saturation levels (only 7% of recordings reached saturation in our cohort). The reasons for this are either due to loudness discomfort or due to time constraints in the clinical routine. The concern here is that the incidence of pre-saturation termination due to loudness discomfort may be higher for cochleae with reduced neural health. This limitation would not be specific to the FI: All the eCAP parameters reported here require that the AGF is accurately represented. Due to the point-symmetry of the sigmoidal function, the saturation amplitude can be identified as long as the inflection point has been captured, which may be more likely when using quasi-continuous stimulation (as implemented in AutoArt) than in classical eCAP-recording paradigms (Estienne et al., 2022). Concordantly, we found that the FI was robust to non-saturating AGFs in that there was no significant difference in average FI-values derived from saturating versus non-saturating AGFs. Furthermore, FI values that were derived from AGFs covering less than 70% of the dynamic range correlated well with FI that were based on AGF that covered at least 70%. We assume that the validity of AGFs irrespective of coverage is due to the applied quality criterion (see discussion above).

A factor that could have influenced our results is a potential surgical trauma resulting from array insertion. Insertion traumas have been reported to temporarily alter the eCAP amplitude growth function (Pfingst et al., 2015). Due to the observed stability over time, we assume that this recovery process was mainly completed at 3 months post-surgery and thus had no systematic effect on our data. Any insertion trauma leading to permanent loss of SGN number or function would, however, add to the variability in neural health, reflected by our study cohort. 

A study in a patient group with supposed low neural health reported elevated numbers of invalid eCAP recordings relative to matched controls (Moyaert et al., 2025). The assumption was supported by the distribution of speech understanding scores in our data (Figure 8), which underrepresented low performers (i.e., <40–70% correct depending on speech task). However, it has been shown that low eCAP amplitudes or even an absence of eCAP responses is not significantly associated with poor speech recognition (Cooper et al., 2020). This is likely due to the fact that the lack of an evaluable eCAP amplitude (i.e., low signal-to-noise ratio) can also be due to low recording quality, resulting in increased noise. This limitation of eCAP-derived measures cannot be resolved at present, as there is currently no method to isolate the impact of low neural health from that of low recording quality. Thus, it may be beneficial to combine the FI with another type of functional marker, such as behavioural psychophysical testing, to offer a more comprehensive evaluation of neural function (for review see, Genitsaridi et al., 2026).

This study had an additional limitation in that no direct neural health measures are available with which to compare the FI. This is a common limitation in the field, as histological preparations from human temporal bones are scarce (for review see, Genitsaridi et al., 2026). The most direct indication that we had are elevated FI in cochleae with auditory nerve hypoplasia (see discussion below). However, this observation is based on only 7 ears in our study cohort, so this finding can only be seen as one indication (among others) that the FI may serve as neural health marker.

The investigation period from the 3^rd^ month to 1^st^ year post-surgery was chosen based on data availability. With increasing time from surgery, a greater fraction of patients tends to miss follow-up visits. This poses a limitation on the interpretation of the outcome measures, as hearing ability with the CI has been found to improve for CI users with postlingual hearing loss within the first year and in some cases even beyond (e.g., Baungaard et al., 2025). However, a recent meta-analysis found no statistically significant improvement after 3 months post-surgery (Ma et al., 2023). As we chose maximal speech-scores for the analyses, the effect of variable improvement throughout the investigation period should be reduced. Furthermore, we found no significant improvement in the sentence-in-noise test from Y1 to Y2 for ears included in this study (paired t-test: t=1.494, df=25, p=0.1478; data not shown).

### Stability Over Time

We found an overall stability of the FI throughout the analysis period. This was important in two regards. First, it enabled us to combine ears that were recorded at different time points. Second, it fits the assumption that SGN degeneration in humans following hair cell loss is a very slow process (as opposed to animal models; for review see Shepherd & Hardie, 2002).

### Group Differences Based on Etiology and Age

We confirmed that the average FI (i.e., mean value over all assessed CI contacts of an ear) is sensitive to demographic factors associated with reduced neural health. Most notably, the FI was elevated in adults over 60 years of age and in children with confirmed auditory nerve hypoplasia. The significantly higher FI in CI users with auditory nerve hypoplasia aligns with reports that eCAP AGFs in paediatric patients with cochlear nerve deficiency have shallower slopes and smaller amplitudes than in children with normal cochlear-nerve anatomy (He et al., 2018). This further corresponds to histopathological observations of reduced SGN counts in this patient group (Nadol Jr 1997).

In CI users with idiopathic etiologies, the mean FI was significantly elevated from 60 years onwards compared to younger users. We also found a significant age-related increase in FI for ears with unspecified genetic cause of hearing loss (i.e., the only common etiology for ears with pre- and postlingual hearing loss in the study population). As no knowledge on the specific genetic mutation was available and likely different types of mutations were included in our small sample population, no further conclusions can be drawn from these results. These findings correspond to other studies, reporting age-related changes in eCAP parameters (e.g., Zamaninezhad et al., 2023) as well as temporal bone studies which reported age-related reductions in SGN counts (Kusunoki et al., 2004) and peripheral processes (Wu et al., 2023).

The variability in FI for different causes of hearing loss was high, likely reflecting the general variability in neural health. This is in agreement with the observation that age-normative SGN loss (as part of neural health) can range from 28% to 100% in patients with hereditary hearing loss (Teufert et al., 2006) and also loss of peripheral processes can vary from 0% to 80% in patients with different causes of hearing loss and sometimes even within one etiology (i.e., Ménière’s disease, Kaur et al., 2026).

### Individual Profiles Along the CI Array

The FI showed site specificity in the sense that it varied across contacts in distinct patterns and between ears. This interpretation is in line with other suggested markers of the neural health profile (DeVries et al., 2016; Pfingst et al., 2004; Wohlbauer et al., 2024; Zhou & Pfingst, 2014). We propose that the sub-population of SGNs most strongly stimulated by a given contact, defines a significant portion of the growth function. At high stimulation levels the growth will not only be affected by neighbouring SGN sub-populations, but also by double-spiking and central shifts in the spike initiation site, which will reduce overall synchrony and thus, eCAP amplitude. Computational models suggest that the eCAP N1P1 amplitude already reaches its peak when only sub-populations of the fibres are excited (Briaire & Frijns, 2005).

We focused our statistical analysis on FI patterns from ears with overall high FI values, as we propose that it is in these cases that the maximal FI along the array is indicative of areas with substantially reduced neural health. In these cases, the maxima were most often located at the most basal contact. The finding is in line with the average FI profiles over the whole population, which showed an overall increase in FI from apical to basal contacts (Figure 3). These observations coincide with areas of aggravated SGN loss in basal (high frequency) areas of the cochlea, as described in temporal bones studies (for review see Shepherd & Hardie, 2002), as well as with degeneration of peripheral processes in the basal half of the cochlea (O'Malley et al., 2024). Still, it is not clear to what degree other factors contributed to the apical-basal gradient observed for the FI, as local anatomy and scalar electrode position may also contribute to neural synchrony, reflected by maximal eCAP amplitudes (Aljehani et al., 2025).

### Correlation With Speech Perception

A common approach to assess whether a measure may serve as neural health marker in human CI users is testing for significant correlation with speech perception. In a first step, we used averaged values (i.e., threshold, slope, maximal amplitude, and FI) over all CI contacts for the correlation with speech outcomes. For the speech scores, we chose the maximum (instead of the mean or median) as many factors may influence performance in the clinical test setting, such as insufficient familiarization with the test material, distraction, time constraints, or fatigue. We concluded that using the highest score achieved across the test sessions would minimize the influence of these factors.

The eCAP threshold was not significantly associated with any of the speech tests. The other three eCAP parameters were correlated with perception of both monosyllabic words and sentences in noise. For both speech tests, the correlation coefficient was slightly higher for the FI than for slope and maximal amplitude, albeit still weak. The overall similarity was unexpected given the fact that the maximal amplitude (other than slope and FI) was dependent on EMD and was also sensitive to changes in phase duration. We conclude that the overall variability in the data (e.g., due to differences in number and position of the stimulating contacts) concealed these differences.

Therefore, we performed a second speech correlation analysis, using a more homogeneous sample of ears with full insertions and selecting a uniform set of ten contacts. Additionally, we chose extreme values of each measure along the array, as we propose that heterogeneous neural health, with focal areas of reduced neural integrity will be more detrimental to speech perception than homogenous degeneration of the same average value. This assumption was confirmed via a comparison to speech correlation analyses with mean values performed for the same sample (Appendix 2).

In line with the initial analysis, the minimal threshold was not significantly correlated with any of the speech-test results, while the outcome measure most strongly correlated with eCAP parameters was the HSM in noise and the eCAP parameter showing strongest relation to speech outcomes was the maximal FI (FI_max_). The correlation coefficient to HSM noise was ∼0.3 for all mean eCAP parameters (highest for FI) and increased when using the extreme values to ∼0.6 (slope_min_ and amplitude_max_) and ∼0.7 (FI_max_). Based on the derived coefficients if determination, the variance in FI_max_ explained about 12% of the variance in the monosyllabic word test. The useability of word recognition tests for assessing neural health markers has been debated: although some studies described a positive correlation between total SGN count and CNC word recognition (e.g., Kamakura & Nadol, 2016), a meta-analysis concluded that monosyllabic word scores were not correlated with SGN survival (Cheng & Svirsky, 2021). However, a recent study found that the variance in estimated number of fibres (as per eCAP recording) explained about 12% of variance in the monosyllabic speech test (Dong et al., 2023). We therefore conclude that the magnitude of the association reported here is in the range expected for an eCAP-derived measures linked to neural health (e.g., 9-12 % in Skidmore et al., 2023). Stronger relations were reported for speech-in-noise tests (Schvartz-Leyzac & Pfingst, 2018; Skidmore et al. 2022, 2023). It has been postulated that in more challenging listening conditions, degraded auditory input from the periphery cannot be sufficiently compensated for by central auditory processes (Skidmore et al., 2023). The variance in FI explained about 55% of the variance in HSM in noise scores. Furthermore, when combined with patient age and duration of hearing loss, the model explained 67% of the variance in speech-in-noise perception, with FI as the single significant predictor.

Commonly, studies that report significant correlations between speech-in-noise scores and electrophysiological markers report coefficients of determination of slightly above 0.3 (Dawson et al., 2025; Dong et al., 2023; Mesnildrey et al., 2020; Schvartz-Leyzac et al., 2025; Skidmore et al. 2022, 2023). This value corresponds well to those described here for mean values of the eCAP parameters, using large sample sizes (N=145). A similarly high coefficient of determination to that of FI_max_ has been described for a different eCAP parameter, the channel-separation index (Scheperle & Abbas, 2015). However, this measure is time consuming (i.e., requiring recording with different combinations of masker and probe) and requires relative assessment of electrode pairs. Furthermore, the correlation with speech perception was observed using an experimental audio processor program rather than the program used in everyday practice. Another approach, resulting in correlation coefficients of about 0.6, is to assess the deviation from the typical relation between eCAP threshold and EMD (Kludt et al., 2025; Long et al., 2014). This approach is, however, dependent on the availability of post-surgical CT scans of sufficient quality. The reported strength of correlation in all three studies (r^2^=0.6) likely reaches the upper limit of how much of the variance in speech understanding is explicable by peripheral factors. Higher values have also been reported, but only using within-subject designs assessing differences between ears (e.g., Schvartz-Leyzac & Pfingst, 2018).

### Independence From Distance to Excitable Structures

In accord with the proposed (partial) normalisation to current spread, both the AGF slope and the FI were independent from the distance to the excitable structures (as estimated by EMD). In agreement with prior studies (Degen et al., 2020; Lee et al., 2021) we found significant elevation in threshold with increasing distance to the modiolus and a significant effect of EMD on maximal eCAP amplitude (Degen et al., 2020; Lee et al., 2021). Of interest, the maximal amplitude peaked at an EMD of around 1.4 mm. To the best of our knowledge, this relation has not been reported before.

### Potential Alternative Implementations of the FI

We established the FI as the input/output ratio at saturation level. As such, a high FI reflects the need to use a high charge level to reach a small maximum output. Recently, the inverse of the FI, the Efficiency Index (max amplitude/charge_max_) has been used as alternative to the FI in a guinea pig model (Meuser et al., 2026). The authors reported cases with zero amplitude in their recordings, which cannot be represented by the FI (i.e., division by zero). This problem is unlikely to arise in the clinical data, where background activity (i.e., recording noise) is high and will determine minimal amplitudes above zero. The FI and the Efficiency Index are interchangeable when being applied for single contacts. However, whenever averages are calculated, the two measures diverge, as the numerator and denominator are weighted differently. The main difference between the two measures is the separation precision between ears when applying the median split approach. Whereas the Efficiency Index separates well the 50% of ears that are assumed to have better neural health, the FI separates well the 50% of ears that are assumed to have reduced neural health. This difference in separation precision has consequences for correlation with speech perception scores. The variance in Efficiency Index values explained about 40% of the variance in speech-in-noise scores (Figure 10C), while the variance in FI explained nearly 55% of these scores (Figure 8C).

As (post-surgical) AGF measurements have to be pre-terminated if the stimulation level is perceived to be too loud, saturation levels are seldom reached. Therefore, recent publications approximated FI at MCL (Garcia & Carlyon, 2025; Wohlbauer & Arenberg, 2025). Whereas this FI_MCL_ could identify simulated cochlear dead regions, it was also significantly affected by EMD. Given that direct comparisons between FI_MCL_ and FI are lacking, it remains speculative which implementation should be recommended under which circumstances. If eCAPs can be recorded with good recording quality, reaching high AGF coverage, we recommend the approach described here. Also in other cases the FI may prove to be more robust than the FI_MCL_, as it is not necessary to record into saturation (see discussion above) and as the MCL has been reported to stabilize only after 6 months post-surgery (Dornhoffer et al., 2025).

### Using the FI_GM_ for Stratification

We propose that a median split approach over all averaged FI values can be used for stratification of CI implanted ears into those with low (<FI_GM_) and high (>FI_GM_) FI. While this approach remains exploratory and the FI_GM_ should not be regarded as a clinically validated cut-off, the dichotomization at FI_GM_ led to similar results as the analyses that were based on continuous FI values. First, all ears with auditory nerve hypoplasia had FI values at or above the FI_GM_ (Figure 6B) and second, the group-level FI values shifted from less than FI_GM_ in younger age groups to greater than FI_GM_ in older age groups (Figure 5). The median split approach was robust to changes in AGF coverage, with more than 80% remaining within the same category (high or low), even when AGF coverage was below 70% of the dynamic range. Thus, we consider the FI_GM_ a useful initial tool to stratify ears for subsequent clinical research. In order to establish a clinically valuable cut-off value for FI-based stratification, validation in independent patient cohorts and across additional research centres is necessary.

### Outlook

A necessary next step in the translation of the FI to clinical practice is to perform prospective cross-over studies. One important aspect is to assess whether the FI can be used as a marker to guide tailored programming strategies for individual CI users. As reviewed by Carlyon and Goehring (2021), individualized audio processor programming in regions of supposed poor neural function (e.g., deactivating selected electrode contacts) has yielded mixed results. Whereby, a recent meta-analysis revealed overall modest effect sizes for re-programming audio processors based on different neural health markers (Lien et al., 2025). If successful, the FI may provide the audiologist with a criterion for electrode selection in clinical audio processor programming (Sander et al., 2023).

## Conclusion

The results presented here are in line with the hypothesis that the FI provides information about neural health profiles, both at the group level and for individual ears. The FI was applicable to a wide range of CI users with different etiologies and demographic backgrounds, including both the paediatric and adult populations and users with pre- and postlingual hearing loss. FI calculation is simple and is based on the standard AGF parameters assessable via clinical software without any additional equipment or time investment needed. We propose that prospective studies should assess the clinical value of the FI. Thereby, the median split approach (FI_GM_) may serve as an initial method to stratify ears, and maxima FI values along the array should be targeted via audio processor re-programming to assess its potential to optimize speech outcomes with the CI.

## Supplemental Material

Supplemental Material - The Failure Index as Marker of the Neural Health Profile in MED-EL CI Users: A Retrospective Analysis Supplemental Material for The Failure Index as Marker of the Neural Health Profile in MED-EL CI Users: A Retrospective Analysis by Wiebke Konerding, Cornelia Batsoulis, Peter Baumhoff, Heval Benav, Lutz Gärtner, Annette Günther, Onhintz de Olano Dieterich, Daniel Schurzig, Stefan Strahl, Jochen Tillein, Sarah Vormelcher, Andreas Büchner, Carolyn Garnham, Andrej Kral in Trends in Hearing

Supplemental Material - The Failure Index as Marker of the Neural Health Profile in MED-EL CI Users: A Retrospective Analysis Supplemental Material for The Failure Index as Marker of the Neural Health Profile in MED-EL CI Users: A Retrospective Analysis by Wiebke Konerding, Cornelia Batsoulis, Peter Baumhoff, Heval Benav, Lutz Gärtner, Annette Günther, Onhintz de Olano Dieterich, Daniel Schurzig, Stefan Strahl, Jochen Tillein, Sarah Vormelcher, Andreas Büchner, Carolyn Garnham, Andrej Kral in Trends in Hearing

## Data Availability

All original data is stored in the database of the German Hearing Center. Pre-processed data, without information on the individual patients, can be shared upon request.*
