# Supplementary material for: The Failure Index as Marker of the Neural Health Profile in MED-EL CI Users: A Retrospective Analysis
Source: Trends Hear. 2026 Aug 20;30:23312165261477505. doi: 10.1177/23312165261477505 (PMC13494344; doi:10.1177/23312165261477505)
Supplement: Supplementary file 1 [file sj-pdf-1-tia-10.1177_23312165261477505.pdf]

Appendix 1 – Patient demographics

Given is the patient number with native language as German, bilingual (including German) or other. The sex (F: female, M: male) is indicated as per self-identification. Per subject, the ear(s) included in analysis are given as right (R), or left (L) and the language status at onset of hearing loss is noted as pre- or post-lingual deafness. The aetiology is usually idiopathic (unknown) and known causes are noted (SHL: sudden hearing loss). For each ear it is noted, if it received any kind of steroid treatment (y: yes) and whether there are disabilities/conditions potentially affecting speech understanding (details on disabilities given in the manuscript; y: yes). The MED-EL implant type is given (all Flex series) and the age at implantation is provided in 20-year bin size (corresponding to Fig. 4). Ears included in the reduced data set (details given in the manuscript) are indicated in bold.

Patient	language	sex	ear	status	aetiology	steroid	disability	implant	age
1	bilingual	F	R	pre	unknown			Flex 28	<20
			L	pre	unknown			Flex 28	<20
2	German	F	L	post	Menière's disease			Flex 24	60-79
3	other	F	R	post	unknown			Flex 28	60-79
4	German	M	R	pre	unknown			Flex 28	<20
			L	pre	unknown			Flex 28	<20
5	German	F	R	post	pertussis			Flex 28	60-79
6	German	F	L	post	traumatic	y		Flex 28	20-39
7	German	M	R	post	unknown			Flex 28	60-79
8	German	M	L	post	Cholesteatoma/SHL			Flex 28	60-79
9	German	M	L	post	unknown			Flex 24	80-91
10	German	M	R	post	unknown			Flex 28	80-91
11	bilingual	F	R	post	SHL			Flex 28	60-79
12	German	M	R	post	unknown			Flex 24	60-79
13	German	F	R	post	Wegener's	y		Flex 28	60-79
14	German	F	R	post	unknown			Flex 28	<20
15	**German**	**M**	**R**	**post**	**SHL**	**y**		**Flex 28**	**20-39**
16	German	F	R	post	unknown			Flex 24	40-59
17	German	M	L	post	unknown			Flex 28	80-91
18	German	F	L	pre	genetic			Flex 28	<20
			R	pre	genetic			Flex 28	<20
19	German	F	L	post	SHL			Flex 24	20-39
20	**German**	**M**	**L**	**post**	**unknown**	**y**		**Flex 28**	**80-91**
21	German	M	R	post	acoustic neuroma			Flex 28	40-59
22	German	F	R	post	unknown			Flex 28	60-79
23	other	M	L	pre	unknown	y		Flex 28	20-39
24	German	M	R	post	unknown			Flex 28	60-79
25	German	M	L	post	unknown	y		Flex 28	80-91
26	bilingual	M	L	pre	genetic			Flex 28	<20
27	German	F	R	post	otitis media			Flex 28	<20
28	other	F	R	post	unknown			Flex 24	20-39
29	German	M	R	post	Menière's disease	y		Flex 28	60-79
30	German	M	L	post	SHL			Flex 28	20-39
31	German	F	R	post	unknown			Flex 24	60-79
32	German	F	R	pre	unknown			Flex 28	<20

32			L	pre	unknown			Flex 28	<20
33	other	M	R	post	unknown	y		Flex 28	60-79
34	German	F	L	post	unknown			Flex 28	40-59
35	other	M	L	pre	unknown			Flex 28	<20
36	German	M	R	post	traumatic			Flex 28	60-79
37	bilingual	F	L	post	SHL	y		Flex 28	60-79
38	German	F	R	post	Menière's disease	y		Flex 28	40-59
39	German	F	R	pre	Cytomegalovirus			Flex 28	<20
40	German	M	L	post	traumatic			Flex 28	40-59
41	German	M	L	pre	hypoplasia			Flex 28	<20
42	German	F	R	post	SHL			Flex 28	40-59
43	German	M	R	post	unknown			Flex 28	40-59
44	German	M	R	post	genetic			Flex 28	40-59
45	German	F	R	post	unknown			Flex 28	20-39
46	**German**	**F**	**L**	**post**	**genetic**	**y**		**Flex 28**	**60-79**
47	German	F	R	post	unknown	y		Flex 28	60-79
48	German	M	L	post	unknown		y	Flex 24	40-59
49	German	F	L	post	unknown		y	Flex 28	80-91
50	bilingual	M	L	pre	genetic			Flex 28	<20
			R	pre	genetic			Flex 28	<20
51	German	M	L	post	genetic			Flex 28	60-79
52	other	F	R	pre	unknown			Flex 28	<20
53	German	M	R	post	unknown			Flexsoft	60-79
54	German	M	R	post	unknown			Flex 28	60-79
55	other	F	R	post	unknown		y	Flexsoft	60-79
56	German	M	R	post	Meningitis			Flex 28	60-79
57	German	F	L	post	SHL			Flex 28	80-91
58	German	M	R	pre	unknown			Flex 28	40-59
59	German	F	R	post	SHL			Flex 28	60-79
60	unknown	F	L	post	SHL			Flex 28	40-59
61	German	F	R	pre	hypoplasia			Flex 28	<20
62	**German**	**F**	**L**	**post**	**unknown**			**Flex 28**	**80-91**
63	**German**	**F**	**R**	**post**	**SHL**	**y**		**Flex 28**	**40-59**
64	**German**	**M**	**R**	**post**	**SHL**	**y**		**Flexsoft**	**40-59**
65	**German**	**F**	**L**	**post**	**unknown**	**y**		**Flex 28**	**60-79**
66	bilingual	F	R	post	Cogan-I-Syndrom			Flex 28	40-59
67	German	F	L	post	SHL			Flex 28	80-91
68	German	F	L	post	unknown		y	Flex 28	40-59
69	German	F	R	post	unknown			Flex 28	80-91
70	German	F	L	post	unknown			Flex 28	40-59
71	other	M	R	pre	unknown			Flex 28	<20
72	German	F	L	post	unknown		y	Flex 28	80-91
73	other	M	R	pre	unknown		y	Flex 28	<20
			L	pre	unknown		y	Flex 28	<20
74	**German**	**M**	**L**	**post**	**unknown**	**y**		**Flex 28**	**40-59**
75	German	M	L	pre	unknown			Flex 28	<20
76	other	F	R	post	unknown			Flex 24	<20

77	German	F	L	pre	LVA-Syndrom			Flex 28	<20
77			R	pre	LVA-Syndrom			Flex 28	<20
78	German	F	R	pre	unknown			Flex 28	<20
79	German	M	L	post	unknown			Flex 28	80-91
80	German	M	L	post	unknown			Flex 28	60-79
81	German	M	R	post	unknown			Flex 28	60-79
82	**German**	**F**	**L**	**post**	**unknown**	**y**		**Flex 28**	**20-39**
			**R**	**post**	**unknown**	**y**		**Flex 28**	**20-39**
83	German	F	L	post	unknown			Flex 28	60-79
84	German	F	R	Post	unknown			Flex 28	60-79
85	German	F	R	Post	unknown			Flex 28	40-59
86	German	M	L	post	SHL			Flex 28	40-59
87	**German**	**F**	**L**	**post**	**SHL**			**Flex 28**	**20-39**
88	**German**	**M**	**R**	**post**	**unknown**	**y**		**Flex 28**	**80-91**
89	German	F	R	pre	unknown			Flexsoft	<20
			L	pre	unknown			Flexsoft	<20
90	German	M	L	pre	unknown			Flex 28	<20
91	German	F	L	post	SHL			Flex 28	20-39
92	German	M	L	post	Menière's disease			Flex 28	60-79
93	bilingual	F	R	pre	unknown			Flex 28	<20
93			L	pre	unknown			Flex 28	<20
94	**German**	**F**	**L**	**post**	**SHL**			**Flex 28**	**40-59**
95	German	M	R	Post	otitis media			Flex 26	60-79
96	bilingual	M	L	post	traumatic		y	Flex 28	40-59
97	other	M	L	pre	hypoplasia			Flex 26	<20
98	other	M	R	pre	unknown			Flex 28	<20
			L	pre	unknown			Flex 28	<20
99	German	F	L	post	SHL			Flex 28	60-79
100	German	M	L	post	unknown			Flex 28	60-79
101	German	F	L	post	unknown			Flex 28	60-79
102	German	F	L	post	unknown	y		Flex 28	40-59
103	**German**	**M**	**L**	**Post**	**unknown**	**y**		**Flex 28**	**60-79**
104	German	F	L	post	unknown			Flex 28	40-59
105	German	F	L	pre	unknown			Flexsoft	40-59
106	other	M	L	pre	unknown			Flexsoft	<20
106			R	pre	unknown			Flexsoft	<20
107	German	M	L	post	unknown			Flex 26	60-79
108	German	M	L	post	SHL			Flexsoft	40-59
109	bilingual	M	L	post	unknown			Flex 28	<20
110	German	M	R	pre	unknown			Flex 28	<20
			L	pre	unknown			Flex 28	<20
111	German	F	L	post	unknown			Flex 28	80-91
112	German	F	L	post	unknown		y	Flex 28	40-59
113	bilingual	M	R	pre	unknown			Flex 28	<20
114	German	M	L	post	SHL			Flex 28	40-59
115	German	F	R	Post	SHL			Flex 28	60-79
116	bilingual	F	L	post	unknown			Flex 28	60-79

117	German	F	L	pre	hypoplasia			Flex 28	<20
118	German	F	R	pre	unknown			Flexsoft	<20
118			L	pre	unknown			Flexsoft	<20
119	bilingual	F	R	pre	unknown			Flex 26	<20
120	bilingual	F	R	pre	unknown			Flex 28	<20
			L	pre	unknown			Flex 28	<20
121	other	F	R	pre	unknown			Flex 28	<20
122	German	F	R	post	Menière's disease			Flexsoft	60-79
123	German	F	R	post	unknown			Flex 28	80-91
124	German	M	L	pre	unknown			Flex 28	40-59
125	German	M	L	post	unknown	y		Flex 28	40-59
126	German	M	R	Post	unknown			Flex 24	60-79
127	German	F	L	post	unknown			Flexsoft	60-79
128	German	F	R	pre	hypoplasia			Flex 28	<20
129	**German**	**M**	**R**	**Post**	**ototoxicity**			**Flex 28**	**60-79**
130	German	F	L	pre	unknown		y	Flexsoft	<20
131	German	M	R	post	unknown			Flexsoft	60-79
132	German	F	L	Post	unknown			Flex 28	60-79
133	other	F	R	pre	unknown		y	Flexsoft	<20
			L	pre	unknown		y	Flexsoft	<20
134	German	M	R	post	acoustic neuroma			Flex 28	60-79
135	German	M	L	post	cortical infarct			Flex 28	60-79
136	German	F	R	post	SHL			Flex 28	40-59
137	German	M	R	Post	tumor			Flexsoft	60-79
138	German	M	L	pre	unknown			Flex 28	<20
139	German	F	L	post	unknown			Flex 28	60-79
140	German	F	L	post	Menière's disease		y	Flex 28	40-59
141	N/A	M	R	pre	unknown			Flex 28	<20
142	German	F	R	pre	hypoplasia			Flex 28	<20
143	German	F	L	post	unknown			Flex 28	60-79
144	German	M	L	post	unknown			Flex 28	80-91
145	bilingual	F	R	pre	Cytomegalovirus			Flex 28	<20
			L	pre	Cytomegalovirus			Flex 28	<20
146	German	F	L	post	unknown			Flex 28	40-59
147	German	F	L	pre	hypoplasia			Flex 28	<20
148	bilingual	F	L	post	chronic mastoiditis			Flex 28	40-59
149	German	M	L	post	Waardenburg syndrome			Flex 28	40-59
150	other	F	L	post	SHL			Flex 28	20-39
151	German	F	L	post	SHL			Flex 26	60-79
152	**German**	**M**	**L**	**post**	**SHL**			**Flex 28**	**60-79**
153	bilingual	F	L	post	unknown			Flex 28	40-59
154	German	F	R	post	schwannoma		y	Flex 28	60-79
155	German	M	L	pre	unknown			Flex 28	<20
156	German	F	L	post	SHL			Flex 28	60-79
157	**German**	**F**	**L**	**post**	**unknown**			**Flex 28**	**40-59**
158	other	F	L	post	Menière's disease			Flex 28	40-59
159	German	M	L	post	SHL		y	Flex 28	60-79

160	**German**	**F**	**L**	**post**	**Menière's disease**			**Flex 28**	**40-59**
161	German	M	R	post	traumatic			Flex 28	40-59
162	**German**	**F**	**R**	**post**	**unknown**	**Y**		**Flex 28**	**60-79**
163	German	F	R	post	unknown			Flex 28	40-59
164	German	F	L	post	unknown			Flex 28	60-79
165	**German**	**M**	**R**	**post**	**unknown**			**Flex 28**	**20-39**
166	German	F	L	post	unknown		y	Flex 28	80-91
167	German	M	L	post	unknown		y	Flex 28	60-79
168	**German**	**M**	**R**	**post**	**SHL**			**Flex 28**	**60-79**
169	German	F	L	post	genetic			Flex 28	20-39
170	**German**	**F**	**L**	**post**	**SHL**			**Flex 28**	**40-59**
171	German	F	R	post	SHL		y	Flex 28	40-59
172	German	M	R	post	ototoxicity/ Menière's			Flexsoft	60-79
173	German	F	L	pre	genetic			Flex 28	<20
			R	pre	genetic			Flex 28	<20
174	other	F	L	post	SHL			Flex 28	60-79
175	German	F	R	post	unknown			Flex 28	60-79
176	**German**	**F**	**R**	**post**	**unknown**	**Y**		**Flex 28**	**20-39**
177	German	F	R	post	unknown			Flex 28	60-79
178	bilingual	M	R	post	unknown			Flex 28	40-59
179	**German**	**F**	**R**	**post**	**unknown**	**Y**		**Flex 28**	**40-59**
180	German	F	L	post	SHL		y	Flex 28	40-59
181	German	F	R	post	unknown			Flex 28	40-59
182	German	M	R	pre	unknown			Flex 28	<20
			L	pre	unknown			Flex 28	<20
183	other	F	L	post	Scarlatina			Flex 28	20-39
184	**German**	**F**	**L**	**post**	**unknown**			**Flex 28**	**40-59**
185	**German**	**M**	**R**	**post**	**Sepsis**	**Y**		**Flex 28**	**20-39**
186	other	M	L	pre	genetic		y	Flex 28	20-39
187	bilingual	M	R	post	unknown			Flex 28	40-59
188	**German**	**F**	**L**	**post**	**Menière's disease**			**Flex 28**	**40-59**
189	other	F	R	post	unknown			Flex 28	20-39
190	German	F	L	post	unknown			Flex 28	<20
191	**German**	**F**	**R**	**post**	**unknown**			**Flex 28**	**40-59**
192	German	M	R	post	unknown			Flex 28	40-59
193	**German**	**M**	**R**	**post**	**SHL**			**Flex 28**	**60-79**
194	**German**	**M**	**R**	**post**	**unknown**			**Flex 28**	**80-91**
195	other	M	L	post	unknown			Flex 28	40-59
196	German	F	R	pre	unknown			Flex 28	<20
			L	pre	unknown			Flex 28	<20
197	German	F	L	post	unknown			Flex 28	20-39
198	German	M	L	post	SHL			Flex 28	40-59
199	German	F	L	pre	unknown			Flex 28	<20
			R	pre	unknown			Flex 28	<20
200	other	F	L	post	unknown			Flex 28	60-79
201	**German**	**M**	**L**	**post**	**unknown**			**Flex 28**	**80-91**
202	German	F	L	post	SHL			Flex 28	60-79

203	German	F	R	post	Menière's disease	y		Flex 28	20-39
204	German	F	R	post	unknown			Flex 28	60-79
205	bilingual	M	L	pre	unknown			Flex 28	<20
			R	pre	unknown			Flex 28	<20
206	bilingual	F	R	pre	unknown			Flex 28	<20
			L	pre	unknown			Flex 28	<20
207	**German**	**M**	**R**	**post**	**unknown**			**Flex 28**	**60-79**
208	German	M	L	post	unknown		y	Flex 28	80-91
209	bilingual	F	L	post	SHL			Flex 28	60-79
210	**German**	**F**	**L**	**post**	**SHL**			**Flex 28**	**60-79**
211	German	M	L	post	unknown			Flex 28	80-91
212	bilingual	M	R	post	traumatic			Flex 28	60-79
213	German	F	L	post	unknown			Flex 28	40-59
214	**German**	**M**	**R**	**post**	**unknown**			**Flex 28**	**60-79**
215	other	F	L	pre	unknown			Flexsoft	<20
			R	pre	unknown			Flexsoft	<20
216	German	F	L	post	unknown			Flex 28	60-79
217	German	M	R	post	SHL			Flex 28	60-79
218	German	M	R	post	unknown			Flex 28	60-79
219	other	F	L	pre	unknown			Flex 28	<20
220	German	M	R	post	SHL			Flex 28	60-79
221	German	M	L	post	SHL			Flex 28	40-59
222	German	M	R	post	unknown			Flex 28	60-79
223	**German**	**M**	**L**	**post**	**SHL**			**Flex 28**	**60-79**
224	**German**	**M**	**R**	**post**	**tumor**			**Flex 28**	**60-79**
225	German	F	L	pre	unknown			Flex 28	<20
226	German	M	R	post	unknown			Flex 28	60-79
227	other	F	L	post	unknown			Flex 28	40-59
228	German	M	R	post	SHL			Flex 28	80-91
229	German	F	L	post	unknown			Flex 28	60-79
230	bilingual	M	R	post	Labyrinthitis			Flex 28	<20
			L	post	Labyrinthitis			Flex 28	<20
231	**German**	**F**	**L**	**post**	**SHL**			**Flex 28**	**20-39**
232	bilingual	M	L	post	SHL			Flex 28	40-59
233	German	F	R	post	unknown			Flex 26	60-79
234	bilingual	M	R	pre	Cytomegalovirus			Flex 28	<20
235	**German**	**M**	**L**	**post**	**SHL**			**Flex 28**	**20-39**
236	**German**	**M**	**L**	**post**	**Menière's disease**			**Flex 28**	**60-79**
237	German	M	R	pre	unknown			Flex 28	<20
238	German	M	L	post	genetic			Flex 26	60-79
239	**German**	**M**	**L**	**post**	**genetic**			**Flex 28**	**60-79**
240	German	F	L	post	SHL			Flex 28	60-79
241	German	F	L	post	unknown			Flex 28	40-59
242	**German**	**F**	**L**	**post**	**unknown**			**Flex 28**	**20-39**
243	German	F	L	post	unknown			Flex 28	20-39
244	bilingual	F	R	pre	unknown			Flex 28	<20
245	German	F	L	pre	Dysplasia/SHL			Flex 26	20-39

246	other	F	R	post	Menière's disease		Flex 28	60-79
247	other	F	L	post	unknown		Flex 28	60-79
248	German	M	L	post	unknown		Flex 28	40-59
249	German	F	L	post	unknown		Flex 28	60-79
250	German	M	R	post	unknown		Flex 28	60-79
251	German	M	R	post	unknown		Flex 28	60-79
252	other	F	L	post	unknown		Flex 28	60-79
253	other	M	R	post	unknown		Flex 28	<20
