# Supplementary material for: The Failure Index as Marker of the Neural Health Profile in MED-EL CI Users: A Retrospective Analysis
Source: Trends Hear. 2026 Aug 20;30:23312165261477505. doi: 10.1177/23312165261477505 (PMC13494344; doi:10.1177/23312165261477505)
Supplement: Supplementary file 2 [file sj-pdf-2-tia-10.1177_23312165261477505.pdf]

Supplement – Speech-in-noise scores had higher correlation with extreme values of the assessed eCAP parameters than with the mean values over the array

As postulated, the correlation coefficients (see Table) and resulting coefficients of determination for the linear regression (see Figure) were always lower when averaging over the whole array (mean) then when using the extreme values (maximum or minimum). The variance in FI explained higher proportions of speech-in-noise scores than any of the other eCAP parameters (i.e., threshold, slope, and maximal amplitude). Thereby, 41% of variance in HSM scores in noise was explained by variance in mean FI and 55% was explained by maximal FI.

Table - Summary of the results of Pearson correlations with HSM speech in noise scores for the same ears as indicated in the manuscript (N=28). Shown are Pearson r- and p-values; significant correlations indicated in bold.

Pearson	FI	threshold	slope	amplitude
EXTREME values				
r	**-0.738**	-0.233	**0.605**	**0.567**
p	**<0.0001**	0.2324	**0.0006**	**0.0017**
Linear R^2	**0.545**	0.054	**0.366**	**0.321**
MEAN values				
r	**-0.643**	-0.104	**0.423**	**0.520**
p	**0.0002**	0.600	**0.0251**	**0.0046**
Linear R^2	**0.414**	0.011	**0.179**	**0.270**

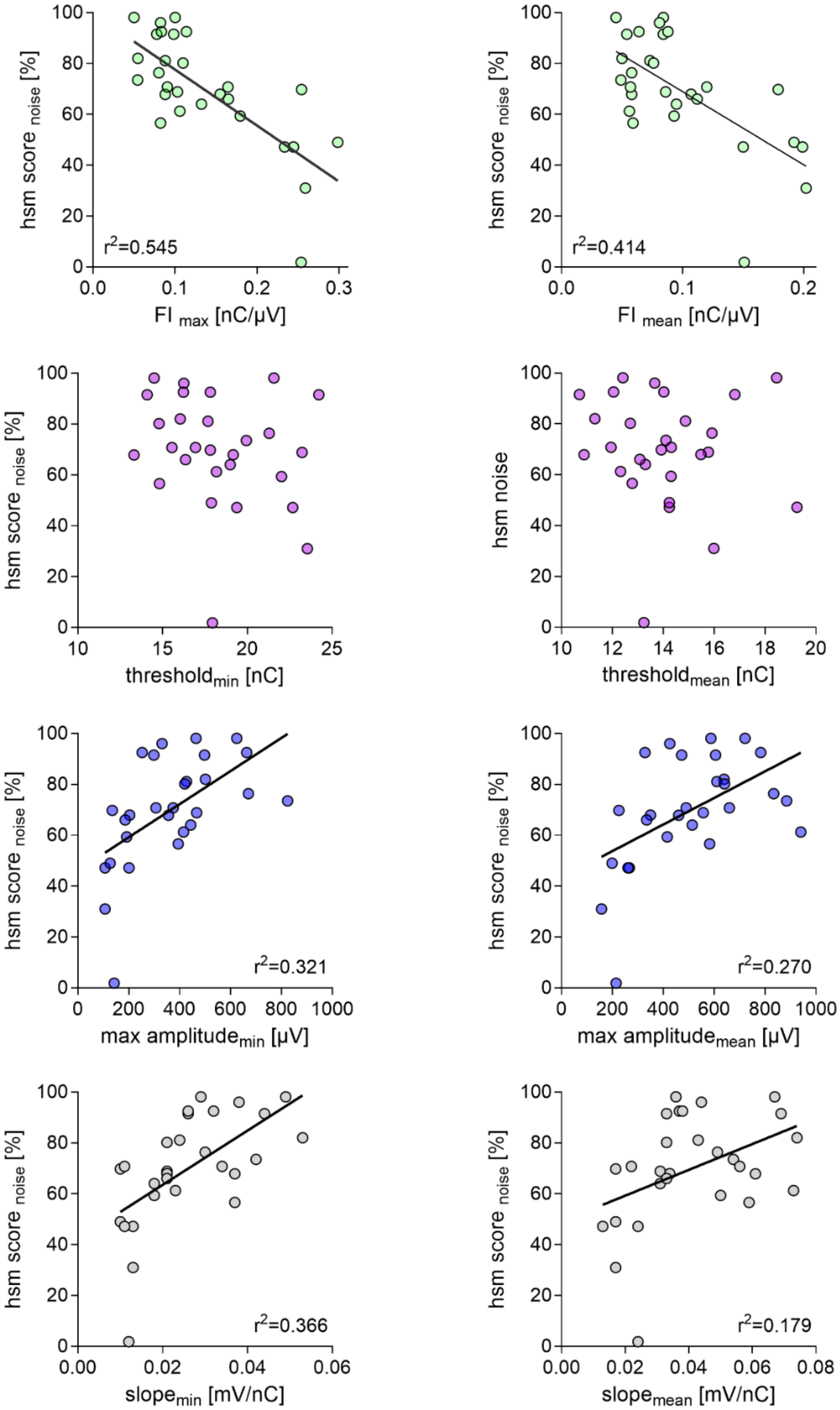

Figure – Relationships between eCAP parameters and speech-in-noise test scores. In addition to the correlations using the extreme values, as reported in the manuscript (left column), the correlations were also assessed using the mean values per array (right column), calculated for the same ears as selected for the initial analyses. Linear regressions with coefficients of determination (r^2) are given for those parameters, showing significant correlation in the Pearson analysis (see Table).
